# Principles of Biocompatible Nanotube Design for High‐Performance Biomaterials

**DOI:** 10.1002/wnan.70075

**Published:** 2026-08-06

**Authors:** Yue Zhong, José A. Lebrón, María L. Moyá, Manuel López‐López, Francisco J. Ostos, Pilar López‐Cornejo

**Affiliations:** ^1^ Department of Physical Chemistry University of Seville Seville Spain; ^2^ Department of Chemical Engineering, Physical Chemistry and Materials Science University of Huelva Huelva Spain

## Abstract

Nanotubes are one‐dimensional tubular structures characterized by nanoscale radial dimensions and micrometer‐scale axial lengths. Their hollow inner cavity confers a high specific surface area and distinctive physicochemical properties, enabling remarkable potential in drug delivery, disease diagnostics, and tissue engineering. Extensive research into their biocompatibility and degradability establishes a solid foundation for further development and rational design. A synthesis of current data across relevant systems, including carbon nanotubes (CNTs), boron nitride nanotubes (BNNTs), titanium dioxide nanotubes (TiNTs), halloysite nanotubes (HNTs), anodic aluminium oxide nanotubes (AAOs), lipid nanotubes (LNTs), and polymeric nanotubes (PNTs), reveals how structural features and physicochemical properties govern biological responses and functional performance. This integration supports a unified “structure–property–bioresponse–function” framework, offering a conceptual roadmap and general design principles to guide the next generation of nanotube‐based biomaterials.

## Graphical abstract

Schematic overview of nanotube‐based biomaterials for biomedical applications.

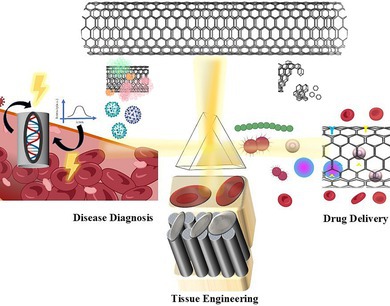

## 1 Introduction

Typically, nanotubes are one‐dimensional tubular materials with nanoscale radial dimensions and micrometer‐scale axial lengths. Their hollow cavities and outer surfaces provide multiple binding sites for drug molecules. Drugs can be non‐specifically adsorbed via hydrophobic and van der Waals interactions, or more specifically bound through hydrogen bonding and electrostatic interactions, significantly enhancing drug loading on both the inner and outer surfaces of nanotubes. Moreover, the pore size and tube length are tuneable parameters that directly influence drug diffusion and release behavior. Consequently, the application of nanotubes in drug loading and release has attracted increasing research attention (Morais 2023).

In addition, inorganic nanotubes feature covalently bonded atoms within their tubular walls, providing high structural strength. When combined with biomacromolecules such as proteins, polysaccharides, or polymers, they can achieve sustained drug release while improving the composite's mechanical properties and the nanotube's biocompatibility. Such composites have demonstrated great potential as tissue scaffolds (Liu et al. 2024). Importantly, various nanotubes exhibit not only tunable pore sizes and high surface areas but also distinct physicochemical and biological properties, with surfaces that can be readily functionalized (Liu et al. 2024; Xu et al. 2022).

For example, carbon nanotubes (CNTs) possess exceptional mechanical strength and outstanding electrical and thermal conductivity, supporting both biomedical reinforcement and sensitive detection of disease‐related biomolecules (Lohani et al. 2025). Because pristine CNTs disperse poorly in aqueous media, surface functionalization is generally required before biomedical use (Kotchey et al. 2013).

Similarly, boron nitride nanotubes (BNNTs) exhibit excellent piezoelectric responsiveness, capable of converting external mechanical energy into localized electrical stimulation. This property makes them ideal for studying the effects of mechanical or electrical signals on cell proliferation, elongation, and differentiation (Lee et al. 2016; Adel et al. 2025).

Titanium dioxide nanotubes (TiNTs) retain the intrinsic hydrophilicity characteristic of oxide‐based materials and present an ordered nanotubular surface topography that resembles the extracellular matrix (ECM). This nanoscale architecture has been associated with enhanced protein adsorption and osteoblast adhesion, which are considered favorable factors for early osseointegration. In vivo studies have reported that, after 4 weeks of implantation of pure titanium discs modified with TiNT surfaces into rabbit tibiae, the bone‐implant bonding strength was nearly nine times higher than that of conventional sandblasted titanium surfaces. Consistently, TiNT‐coated implants exhibited markedly greater bone‐implant contact areas and increased new bone formation along the nanotube‐covered regions (Bjursten et al. 2010).

When inert materials come into contact with living tissues, the host may elicit responses such as toxicity, irritation, teratogenicity, and localized inflammation; accordingly, biocompatibility is a mandatory consideration (Bianco et al. 2011). Although CNTs and BNNTs produced using current methods can satisfy specific biomedical functions, such as imaging or electrical conduction, they often cannot simultaneously and fully meet biocompatibility requirements. Therefore, selecting appropriate nanotube structures and applying targeted surface treatments or chemical modifications to improve biocompatibility while retaining the required functions has become a major focus of current research (Maghimaa et al. 2024).

Furthermore, after nanotubes enter biological environments in vivo, enzymes, acidic or basic species, and reactive radicals may initiate material degradation through catalytic, hydrolytic, or oxidative mechanisms (Bianco et al. 2011; Maghimaa et al. 2024; Zhang et al. 2024). Unlike biocompatibility, degradability is not a uniform mandatory requirement for every application; nevertheless, it should be regulated through material selection, structural design, or modification according to the intended use and performance requirements (Alkhamis et al. 2025). Thus, a comprehensive understanding and rational control of biocompatibility and degradability are essential for the biomedical application of nanotubes. Beyond CNTs, BNNTs, and TiNTs, other systems with considerable application potential include inorganic halloysite nanotubes (HNTs) and anodic aluminium oxide nanotubes (AAOs), as well as organic lipid nanotubes (LNTs) and polymeric nanotubes (PNTs).

This review aims to integrate current knowledge on biocompatibility, degradability, and biomedical applications of various nanotubes within a unified framework that links structural design, physicochemical properties, biological responses, and biomedical functions. This “structure–property–bioresponse–function” correlation provides general design principles for advancing the biomedical development of nanotube‐based materials.

## 2 Biocompatibility of Nanotubes

The biocompatibility of nanotubes is not determined solely by material category, but is jointly influenced by surface chemistry, morphology, length, purity, dispersion and aggregation state, and exposure dose. Current evidence indicates that surface treatment and chemical modification are among the principal approaches for improving nanotube biocompatibility and dispersibility. For example, introducing hydrophilic functional groups such as carboxyl groups (—COOH) onto CNT surfaces can improve dispersion stability and, under specific experimental conditions, reduce damage to cell viability and membrane integrity (Heister et al. 2010; Zhang et al. 2012). Similarly, surface functionalization of BNNTs can improve their surface reactivity and bio interfacial properties (Zhi et al. 2005; Ciofani et al. 2012). In addition, poly(ethylene glycol) (PEG), proteins, nucleic acids, and other natural or synthetic macromolecules can modify nanotube surfaces through covalent attachment or noncovalent adsorption, thereby improving dispersion stability in aqueous and biological media. Appropriately constructed hydrophilic barriers such as PEG can also reduce nonspecific protein adsorption and improve cytocompatibility and overall biological tolerance (Heister et al. 2010; Zhang et al. 2019). To facilitate comparison across materials, Table 1 summarizes four major classes of surface‐functionalization strategies currently used to improve nanotube biocompatibility, together with their general mechanisms and principal limitations.

**TABLE 1:** Typical surface functionalization strategies for improving the biocompatibility of nanotube‐based biomedical materials.

Surface functionalization strategy	Main biocompatibility improvement	General mechanism	Main limitation
Reactive functional group modification, such as hydroxyl, carboxyl, and amino groups	Improved hydrophilicity, dispersibility, molecular coupling capacity, cytocompatibility, hemocompatibility, and degradability	Increased surface polarity, reduced aggregation, and creation of reactive or defect‐containing sites	Over‐functionalization may damage nanotube structure or alter intrinsic properties
Hydrophilic, polymeric, natural, or biomimetic coating	Reduced protein adsorption, biofouling, macrophage recognition, inflammatory activation, and blood‐contact activation	Formation of hydrated steric or biomimetic interfacial barriers	Coating instability or excessive shielding may impair nanotube functions
Biofunctional molecule modification, such as anticoagulant, anti‐inflammatory, or adhesive molecules	Improved blood compatibility, reduced inflammation, reduced foreign‐body response, or enhanced tissue integration	Introduction of specific biological activities at the nanotube–biointerface	Biological activity depends on molecular stability, orientation, density, and retention
Surface ionization or plasma‐assisted hydrophilization	Improved surface hydrophilicity, aqueous dispersibility, and subsequent modification capacity	Plasma‐induced activation and introduction of polar surface groups	Excessive treatment may introduce structural defects or alter adsorption behavior

*Note:* This table focuses on surface‐functionalization strategies primarily intended to improve biocompatibility rather than to enhance nanotube‐specific functional performance.

The first class comprises reactive functional‐group modifications, including hydroxylation, carboxylation, and amination. These strategies improve biocompatibility mainly by changing surface polarity, reducing aggregation, and providing reactive sites for subsequent molecular coupling (Yue et al. 2023). Oxygen‐containing functional groups such as hydroxyl and carboxyl groups can improve the aqueous dispersibility of CNTs and may introduce defect‐associated sites that facilitate oxidative enzymatic degradation (Bianco et al. 2011). Similar principles can be applied to other nanotube systems, including covalently functionalized BNNTs, in which reactive groups improve surface reactivity and biointerfacial properties (Ciofani et al. 2012). However, such functionalization is not invariably beneficial: excessive oxidation or over‐functionalization may damage the tubular wall, disturb the surface‐charge balance, or compromise intrinsic mechanical and electrical properties.

The second class comprises hydrophilic, polymeric, natural, or biomimetic coatings. Their principal role is to reduce direct contact between the nanotube surface and biological components and to suppress nonspecific adsorption of proteins, inflammatory mediators, and other biomolecules by forming hydrated steric barriers or biomimetic interfacial layers. For example, polysaccharide‐ or protein‐like modifiers can improve the dispersion stability of oxidized multi‐walled carbon nanotubes (MWCNTs), whereas phosphatidylcholine modification can reduce nonspecific protein adsorption and improve blood‐contact behavior (Bi et al. 2012; Larasati et al. 2022). Coating stability nevertheless remains an important limitation, particularly for physically adsorbed coatings. Excessive coverage may also block nanotube openings or lumens and thereby impair drug loading, molecular adsorption, and release functions.

The third class comprises bio functional‐molecule modification. Its purpose is not merely to increase hydrophilicity, but to introduce specific biological activities at the nanotube–bio interface. Anticoagulant, anti‐inflammatory, or adhesive molecules can regulate coagulation responses, inflammatory signaling, cell adhesion, or tissue integration, respectively. For example, after polyethyleneimine (PEI) is attached to a CNT surface, further grafting of heparin onto the PEI layer can improve hemocompatibility (Murugesan et al. 2006). The practical effect of this strategy, however, depends on molecular stability, orientation, surface density, and retention; consequently, the same molecule may not produce consistent biological outcomes when different grafting methods or loading conditions are used.

The fourth class comprises surface ionization or plasma‐assisted hydrophilization. This strategy can modify nanotube surface chemistry relatively directly without forming a separate coating layer. Argon‐plasma treatment, for example, can activate CNT surfaces and alter their surface properties (Yan et al. 2005), whereas reactive‐plasma treatment of TiO_2_ nanotubes can increase and prolong surface hydrophilicity, thereby supporting biocompatibility and subsequent modification (Kunrath et al. 2020). Excessive treatment may nevertheless introduce structural defects and alter protein adsorption and interfacial biological responses; treatment intensity and duration must therefore be matched to the material's functional requirements.

Building on these general strategies, the biological response to CNTs remains strongly dependent on nanotube type, length, purity, and surface charge. Pristine CNTs readily aggregate and can be cytotoxic (Orecchioni et al. 2014; Aoki and Saito 2020). Long multi‐walled carbon nanotubes (MWCNTs; > 20 μm) have been associated with carcinogenic risk, whereas MWCNTs shorter than 1 μm did not produce mesothelioma after 24 months of exposure (Poland et al. 2008; Muller et al. 2009). Although surface functionalization generally reduces toxicity (Sun et al. 2017; Tan et al. 2021; Kumar et al. 2023), its effects depend on surface chemistry: anionic groups such as —COOH are associated with weaker inflammatory responses, whereas strongly cationic polyethyleneimine coatings can trigger cytokine release and pulmonary fibrosis in mice (Orecchioni et al. 2014).

In vitro experiments indicate that pristine BNNTs exhibit greater cytotoxicity than CNTs, largely due to intracellular accumulation (Horvath et al. 2011) and differences in morphology, synthesis method, dosage, and length. BNNTs of 1.5 μm show negligible cytotoxicity (Ciofani et al. 2014), whereas those of approximately 10 μm display evident toxicity. In vivo, inhalation of 40 μg unmodified BNNTs (0.1–0.3 μm in length) by C57BL/6J mice induces acute pulmonary inflammation (Kodali et al. 2017). In contrast, chitosan‐coated BNNTs (~1.5 μm) administered intravenously to rabbits at 10 mg/kg caused no detectable toxicity (Ciofani, Danti, et al. 2013).

TiNTs are chemically stable under physiological conditions and generally regarded as biologically inert; they have been used in several US Food and Drug Administration (FDA)‐approved biomedical applications, including orthopedic implant coatings and dental restorations (Shi et al. 2013). Pristine TiNTs typically support cell adhesion and proliferation without eliciting severe immune rejection (Cao et al. 2024). After implantation, TiNT‐coated titanium enhances osseointegration and bone‐implant interfacial strength, with no chronic inflammation or fibrous encapsulation observed (Liu et al. 2021). Doping TiNT surfaces with bioactive elements or combining them with biomolecules further improves cell compatibility (Cao et al. 2024). However, in vitro studies have reported that at high concentrations (0.5–1.0 mg/mL), TiNTs can induce oxidative stress and DNA damage in human dermal fibroblasts (Mohamed et al. 2017).

HNTs (~2 μm in length) exhibit minimal effects on the viability of HeLa and MCF‐7 cells at low concentrations, with approximately 70% viability remaining at 75 μg/mL (Vergaro et al. 2010). These differences arise from intrinsic structural and compositional variability associated with the geological origin of halloysite. HNT‐U and HNT‐S differ in their lumen diameter, external surface roughness, and degree of tubular ordering, all of which influence cellular interactions. Moreover, variations in the Al/Si ratio, hydration state of the inner aluminium layer, and the amount of natural mineral impurities (e.g., Fe^3+^, Ca^2+^, Mg^2+^) can alter surface charge density and protein adsorption behavior. Such structural and chemical disparities modulate dispersion stability, cellular uptake kinetics, and membrane interaction strength, ultimately leading to distinct cytotoxicity profiles among HNTs from different deposits. However, at 600 μg/mL and 72 h exposure, both HNT‐U and HNT‐S induce markedly higher cytotoxicity in HeLa cells, and HNTs similarly show increased toxicity toward HepG2 cells (Lazzara et al. 2023). Surface modification substantially enhances HNT biocompatibility (Liao et al. 2023).

AAO nanoporous membranes are generally non‐toxic to cells (La Flamme et al. 2007). When intravenously injected, AAOs are primarily phagocytosed by the mononuclear phagocyte system, accumulating in the liver and spleen. No acute toxicity was observed within 28 days at low and medium doses, whereas moderate hepatotoxicity occurred at high doses. Subcutaneous implantation induced localized granulomatous inflammation (Wang, Zinonos, et al. 2017). Dissolved aluminium ions released through wear can complex with citrate or lactate, penetrate biological membranes, and cross the blood–brain barrier (BBB), potentially causing neurotoxicity (Bryliński et al. 2023). PEG surface modification improves metabolic clearance and biosafety, reduces inflammatory reactions, and accelerates systemic elimination (Domagalski et al. 2021).

LNTs formed by self‐assembly of natural phospholipids possess excellent biocompatibility. Upon entering the bloodstream, they may undergo immune recognition and opsonization (Lila et al. 2013). Modulation of surface charge density (Masuda and Shimizu 2004) and protein adsorption behavior (Palchetti et al. 2016), or modification with PEG and other polymers, can reduce clearance by the immune system while simultaneously enhancing tumor accumulation and therapeutic efficacy (Liu et al. 2023; Sui et al. 2023).

The flexible architecture of PNTs minimizes mechanical damage to cells (Newland et al. 2018), while surface hydration layers inhibit protein adsorption (Suk et al. 2016). In vitro 3‐(4,5‐dimethylthiazol‐2‐yl)‐2,5‐diphenyltetrazolium bromide (MTT) and agar‐diffusion assays using mouse fibroblasts demonstrated excellent cyto‐ and hemocompatibility, with negligible haemolysis or acute immune response (Shen et al. 2019). However, lactic acid, a degradation‐product of poly(lactic acid) (PLA) and poly(lactic‐co‐glycolic acid) (PLGA), may induce local inflammation (Zhou et al. 2022).

Overall, surface functionalization can improve nanotube dispersibility and biocompatibility to varying degrees by modifying surface polarity, constructing interfacial barriers, introducing specific biological activity, and increasing capacity for subsequent modification. Its effectiveness is nevertheless constrained by modification stability, surface density, structural damage, and functional shielding. Nanotube biocompatibility should therefore still be understood as the combined outcome of surface chemistry, morphology, purity, and dispersion state, forming the first level of linkage between material structure and biological response.

## 3 Degradability and Metabolic Behavior of Nanotubes

The biodegradation of nanotubes refers to the gradual reduction or disappearance of their structural dimensions through a series of chemical reactions and physical transformations within biological environments. This process is influenced by both the intrinsic physicochemical properties of the nanotubes and the specific biological environment, such as the presence of catalytic enzymes and local pH. Among these factors, the chemical composition of nanotube material plays the most decisive role.

For instance, nanotubes composed of elements already in their highest oxidation states, such as Ti^4+^ in TiNTs and Si^4+^ in HNTs, cannot undergo oxidative degradation. Because of their low reactivity toward acids, these inorganic nanotubes also exhibit minimal dissolution or corrosion under physiological conditions. In contrast, nanotubes containing low‐valence carbon atoms are susceptible to oxidation. Studies have shown that CNTs bearing oxygen‐containing functional groups can be oxidatively degraded by hydrogen peroxide in the presence of peroxidase enzymes (Bianco et al. 2011). Unlike inorganic nanotubes, organic systems such as LNTs or PNTs can typically undergo complete degradation and metabolic clearance. An exception is PEG‐based nanotubes, which are highly stable and resistant to enzymatic or hydrolytic degradation.

CNTs degrade slowly and are difficult to excrete, often accumulating extensively within the mononuclear phagocyte system, possibly due to atypical recognition by T‐cell immunoglobulin and mucin domain‐containing protein 4‐positive (TIM4^+^) macrophages (Omori et al. 2021). Plant peroxidases such as horseradish peroxidase can progressively “cut” oxidized single‐walled carbon nanotubes (SWCNTs) in the presence of hydrogen peroxide, leading to near‐complete degradation within 10 days and producing short oxygenated polyaromatic hydrocarbons or CO_2_ (Bianco et al. 2011). SWCNTs degrade more readily than MWCNTs; shorter CNTs with hydroxyl or carboxyl groups or other surface functionalities are more susceptible to enzyme‐catalyzed oxidation than longer CNTs (Yang and Zhang 2019).

BNNTs are chemically inert and resistant to enzymatic or hydrolytic degradation in vivo. Nevertheless, chitosan‐modified BNNTs exhibit improved biocompatibility and can be partially cleared from the bloodstream (Kodali et al. 2017). When gum arabic‐coated BNNTs (200 μg/g) were injected into planarians, the nanotubes displayed good compatibility but also showed a risk of hepatic and splenic accumulation (Salvetti et al. 2015).

TiNTs display high chemical stability and are resistant to biodegradation or dissolution under physiological conditions because of their extremely low solubility in water (Yu et al. 2011). Consequently, TiNT coatings on implants can maintain their morphology and function over extended periods, showing minimal corrosion or dissolution in body fluids. Intravenous injection of TiO_2_ nanoparticles (1 mg/kg) in mice resulted in preferential accumulation in the liver and spleen (mononuclear phagocyte system) with pronounced retention (Disdier et al. 2015).

Few studies have investigated the in vivo metabolism of HNTs. Based on their chemical composition, Al_2_Si_2_O_5_(OH)_4_·nH_2_O (Chiew et al. 2014; Ma et al. 2017), and kaolinite‐like tubular morphology (Ganapathy et al. 2022), aluminium and silicon exist in crystalline form within the nanotubes, rendering them resistant to acid degradation in biological environments.

AAO nanotubes are stable alumina ceramics that exhibit extremely low degradability under physiological conditions (Domagalski et al. 2021). Mechanical wear may cause partial dissolution, releasing Al^3+^ ions that can complex with citrate or lactate, facilitating membrane transport and even BBB penetration, which may result in neurotoxicity (Bryliński et al. 2023).

LNTs, formed by the self‐assembly of natural phospholipids, are readily biodegradable under physiological conditions. They undergo enzyme‐catalyzed metabolic degradation, leading to complete decomposition into naturally occurring biomolecules (Chai and Yao 2013).

The degradation behavior of PNTs depends strongly on polymer chemistry, molecular weight, crystallinity, structural dimensions, and copolymer composition. Among common biodegradable aliphatic polyesters, PLGA generally degrades faster than PLA, whereas PCL degrades more slowly; however, this relative order may vary with formulation and material structure. Within PLGA systems, the lactic acid‐to‐glycolic acid ratio is a major determinant of degradation. For example, PLGA 50:50 was reported to degrade completely within 6–8 weeks because of its relatively high glycolic acid content and hydrophilicity, whereas PLGA 85:15 required approximately 26 weeks (Ramchandani et al. 1997). The degradation products of PLGA, namely lactic and glycolic acids, and those of PLA, primarily lactic acid, can enter endogenous metabolic pathways, including the tricarboxylic acid (TCA) cycle (Patel et al. 2010). PCL degrades much more slowly because of its greater hydrophobicity and relatively high crystallinity; complete degradation in vivo may require up to 2 years, with 6‐hydroxycaproic‐acid‐related products undergoing gradual metabolic processing and clearance (Roberson 2022). PEG should not be included in the same biodegradation ranking because it is largely resistant to enzymatic and hydrolytic degradation. Instead, the in vivo fate of unbound linear PEG chains is primarily governed by molecular‐weight‐dependent glomerular filtration and tissue retention (Baumann et al. 2019). PEG molecules below 20 kDa are cleared more readily, whereas those above approximately 30–40 kDa may show prolonged tissue retention (Baumann et al. 2019). Therefore, PEG chain length should be carefully controlled, or cleavable linkages should be incorporated into PEG‐containing nanotube carriers to facilitate eventual clearance.

Overall, the degradability and metabolic fate of nanotubes are primarily dictated by their bond type and chemical reactivity, linking their intrinsic physicochemical stability to long‐term biological behavior.

The evidence above demonstrates substantial differences among nanotube systems in degradation rate, metabolic processing, and residual form. To compare these differences within a unified framework, Table 2 provides a semiquantitative summary of the relative degradation tendencies and major in vivo processing pathways of representative nanotube biomaterials under physiological conditions.

**TABLE 2:** Semiquantitative ranking of degradation and major metabolic/clearance pathways of representative nanotube biomaterials under physiological conditions.

Nanotube system	Relative degradation score	Major in vivo processing and ultimate fate
LNTs	7	Entry into endogenous lipid metabolism after disassembly
PNTs	6–4	PLA/PLGA: endogenous organic‐acid metabolism; PCL: slow small‐molecule metabolism/renal clearance; PEG: molecular‐weight‐dependent renal excretion or tissue retention
CNTs	3	Oxidative fragmentation; macrophage uptake and retention in the mononuclear phagocyte system
HNTs	2	Limited transformation; phagocytosis of residues and tissue retention
AAOs	2	Formation of stable particles; post‐phagocytic retention and potential aluminium‐related release
TiNTs	2	Long‐term surface stability; local retention of detached particles or residual persistence after phagocytosis
BNNTs	1	Long‐term persistence of the nanotube body; not clearly established metabolic or excretory pathway

*Note:* Scoring note: 7 = fastest degradation/lowest in vivo persistence; 1 = slowest degradation/highest in vivo persistence. This score is a semiquantitative synthesis of the relative degradation tendencies of the nanotube systems reviewed here and should not be interpreted as an absolute degradation‐rate constant.

The biosafety of nanotube degradation products should be evaluated according to whether the resulting species can enter physiological metabolic or excretory pathways, or instead persist as durable particles, ions, or incompletely transformed residues.

Lipid nanotubes have a clear advantage in this respect: the principal components remaining after degradation or disassembly are still predominantly lipid molecules and can generally be incorporated into endogenous lipid metabolism, transport, or membrane‐renewal processes (Kyriakides et al. 2021). Compared with persistent inorganic residues or poorly metabolized carbon‐based fragments, lipid‐nanotube degradation products therefore generally exhibit better biocompatibility and a lower risk of long‐term persistence. This advantage nevertheless depends on the site of degradation, release rate, and local lipid‐processing capacity; high doses, rapid local release, or insufficient processing capacity in specific tissues may still lead to local accumulation, immune recognition, or off‐target distribution.

Polymeric nanotubes generally exhibit good biocompatibility and designable degradation behavior, while the safety of their degradation products depends mainly on polymer type and the corresponding metabolic intermediates. PLA‐ and PLGA‐based nanotubes primarily degrade into lactic‐acid‐ and glycolic‐acid‐related products. These small molecules can enter endogenous metabolic pathways and are therefore generally amenable to physiological processing (da Silva et al. 2018; Martins et al. 2018). Because lactic acid and glycolic acid are acidic products, however, high doses, rapid degradation, or insufficient local buffering may still cause a local decrease in pH or mild inflammatory irritation. PCL‐based nanotubes degrade more slowly and mainly generate 6‐hydroxycaproic‐acid‐related products. Their short‐term acidic burden is relatively low, but their longer degradation period requires greater emphasis on in vivo residence time, sustained exposure, and delayed clearance. PEG‐based systems differ from typical degradable polyesters: rather than generating well‐defined small‐molecule metabolic intermediates, they mainly exhibit molecular‐weight‐dependent clearance of PEG chains or fragments (Baumann et al. 2014). Low‐molecular‐weight PEG is more readily excreted by the kidneys, whereas higher‐molecular‐weight or structurally complex PEG fragments may require longer processing through the liver, spleen, or mononuclear phagocyte system. Overall, polymeric‐nanotube degradation products generally have a favorable safety basis, but the risks of local accumulation or delayed clearance should still be assessed in relation to polymer type, degradation rate, dose, PEG molecular weight, and the presence of marked hepatic or renal dysfunction.

For carbon nanotubes, the safety of degradation products depends mainly on whether intact tubular structures can be progressively converted into shorter and smaller carbonaceous fragments that can be processed by phagocytic systems. CNT synthesis and growth inherently generate a degree of structural defects, edge sites, and surface irregularities that may serve as starting points for oxidative or enzyme‐assisted degradation (Yang et al. 2012). Compared with highly stable inorganic nanotubes, CNTs have some potential for progressive fragmentation; their biocompatibility should therefore not be simplistically classified as entirely unfavorable. Their degradation intermediates can be understood as carbonaceous fragments detached from, or generated through oxidation of, the nanotube framework. When these fragments are further shortened and processed by phagocytic cells, their long‐term hazards may be lower than those of intact, long, or highly aggregated CNTs. Nevertheless, CNT degradation is generally slow and incomplete and is influenced by wall number, length, defect density, dispersion state, and purity. Multi‐walled, long, or aggregated CNTs in particular may persist for prolonged periods in the mononuclear phagocyte system (Yang et al. 2012). Accordingly, the key issue in evaluating CNT degradation‐product safety is not whether conventional chemical toxicity is generated, but whether intact nanotubes can progressively fragment into shorter, smaller, clearance‐compatible carbonaceous species and whether residual fragments induce chronic inflammation, granuloma formation, fibrosis, or other long‐term responses because of their size, morphology, aggregation state, or residual impurities.

For nanotube systems with low degradability, including HNTs, AAOs, TiO_2_ nanotubular arrays, and BNNTs, the biosafety question is closer to “residual‐material safety” than to the classical safety of metabolic products. HNTs are relatively more capable of undergoing limited transformation. Although their natural origin offers certain application advantages, their mineral composition and impurities cannot be completely controlled; safety assessment should therefore focus on aluminosilicate‐framework fragments, surface‐transformation products, mineral impurities, tissue accumulation, and long‐term inflammatory responses. AAOs and TiO_2_ nanotubular arrays exhibit still lower degradability. Their principal concern is not conventional metabolic toxicity, but the formation of stable particles after wear, fracture, or detachment, leading to local retention, residual persistence after phagocytosis, and chronic inflammation (Crisponi et al. 2013; Luo et al. 2020). In contrast, the boron nitride framework of BNNTs is the most stable and lacks clearly established in vivo degradation and metabolic pathways (Merlo et al. 2018).

Overall, nanotube degradability and metabolic fate are primarily dictated by bond type and chemical reactivity, while the ability of degradation products to enter predictable metabolic or excretory pathways further determines their long‐term persistence risk. The relative ranking in Table 2 does not represent absolute degradation rates; rather, it integrates intrinsic material stability, in vivo processing, and potential persistence within a common comparative framework.

## 4 Nanotube‐Related Properties, Mechanistic Pathways, and Pathobiological Consequences

The preceding two sections summarized the biological performance of different nanotube systems from the perspectives of biocompatibility and degradability/metabolic behavior. Surface chemistry, tubular geometry, dispersion and aggregation state, and material persistence are not independent, however, but jointly determine a continuous process from initial biological contact to ultimate in vivo fate. Surface modification, shape, length, and aggregation state can substantially alter cellular responses and in vivo behavior (Liu et al. 2013). Because the numerous parameters and their combinations cannot be exhaustively addressed within a limited space, this section selects these four representative dimensions to establish an analytical sequence from nanotube‐related factors, through mechanistic pathways, to dominant in vivo consequences (Table 3). The framework can be used both to explain how material design is translated into biological responses and, when a specific in vivo consequence is observed, to indicate in reverse the mechanistic processes and material factors that may be abnormal.

**TABLE 3:** Mechanistic links between nanotube‐related properties and pathobiological consequences.

Nanotube‐related factor	Mechanistic pathway	Dominant in vivo consequence
Surface chemistry	Biointerface formation and host recognition	Hemocompatibility disturbances and acute inflammatory reactions
Tubular geometry, aspect ratio, and rigidity	Geometry‐dependent cellular interaction and incomplete clearance	Foreign‐body reaction and fibrotic encapsulation
Dispersion and aggregation state	Aggregation‐driven redistribution and phagocytic sequestration	Altered biodistribution and off‐target organ accumulation
Degradation and residual fate	Persistence‐dependent clearance and cumulative exposure	Prolonged tissue persistence and delayed clearance

Surface chemistry influences early host recognition by regulating the bio interface formed after nanotubes enter the body. Surface charge, wettability, and functional groups can alter protein adsorption, cellular interactions, and platelet behavior (Liu et al. 2013; Cheng et al. 2018). These effects mainly involve two approaches: forming hydration or steric barriers that reduce nonspecific biomolecular adsorption (Martin and Kohli 2003) and selectively enriching endogenous proteins such as albumin and apolipoproteins to form a transient protective protein corona (Cai and Chen 2019). Both approaches may improve hemocompatibility over the short to intermediate term, but their effectiveness is limited by coating stability, protein exchange, and interfacial reconstruction (Cai and Chen 2019). Once the protective interface becomes unstable, reactive sites may be re‐exposed, thereby promoting complement‐, platelet‐, and coagulation‐related responses (Cai and Chen 2019). Specific surface‐functionalization states may also enhance inflammatory‐pathway activation and cytokine release in monocytes (Pescatori et al. 2013). Specific surface‐functionalization strategies are summarized in Table 1 and are not repeated here.

Tubular geometry, aspect ratio, and rigidity jointly determine whether nanotubes can retain the functional advantages of their tubular architecture while remaining amenable to cellular processing. Appropriate control of nanotube length and morphology may increase the likelihood of complete engulfment and processing by phagocytes. Compared with curved or entangled materials, long and straight nanotubes are more likely to induce frustrated phagocytosis, reduced phagocytic capacity, and increased release of tumor necrosis factor‐α (TNF‐α) and reactive oxygen species (Liu et al. 2013; Brown et al. 2007). Geometric parameters cannot be reduced without limit, however. Excessive shortening may compromise the structural advantages required for lumen‐based loading and directional transport, whereas insufficient mechanical stability may cause bending, collapse, or premature fracture and thereby diminish the functional value of the tubular architecture (Martin and Kohli 2003). Conversely, when nanotube length, aspect ratio, or rigidity exceeds cellular processing capacity, complete engulfment and clearance may be restricted and accompanied by sustained membrane contact, local retention, and inflammatory‐mediator release (Brown et al. 2007; Zent and Elliott 2017). Macrophages, foreign‐body giant cells, and fibroblasts may subsequently accumulate around the material, ultimately producing a local foreign‐body reaction and fibrotic encapsulation (Chandorkar et al. 2019).

Dispersion and aggregation state determine the relationship among colloidal stability, local enrichment, and functional output in vivo. Ideally, nanotubes should maintain appropriate interparticle spacing through electrostatic repulsion (Overbeek 1977) and avoid irreversible aggregation through steric hindrance or interfacial hydration, while retaining the capacity for delivery to and enrichment within the target region (Cai and Chen 2019; Gong et al. 2014). A highly dispersed system that cannot achieve effective target‐site deposition or cellular uptake may still have limited functional output. Conversely, when interparticle attraction predominates, aggregation increases the effective biological size and promotes sequestration by the mononuclear phagocyte system (Cai and Chen 2019; Chen et al. 2004). The resulting distributional shift may divert the material from its intended target, ultimately leading to altered biodistribution and off‐target organ accumulation (Cai and Chen 2019; Al‐Jamal et al. 2012).

The key objective of degradation design is to match material lifetime to the functional period, so that the necessary structure and performance are retained until the intended function has been completed and degradation proceeds thereafter (Li et al. 2020). Excessively rapid degradation may impair nanotube‐mediated transport and release, whereas excessively slow degradation may prolong tissue exposure and increase the clearance burden (Li et al. 2020; Zhang et al. 2019). Degradation rate and residual form are jointly influenced by surface chemistry, structural defects, and wall architecture, which in turn determine whether degradation products can be effectively processed and cleared (Zhao et al. 2011; Allen et al. 2009). Degradation design should therefore also control the residual form and biological risk of the resulting products. Persistent parent materials or residues may lead to prolonged tissue persistence and delayed clearance (Yang et al. 2008). The degradation tendencies, metabolic pathways, and residual risks of different nanotube systems are compared in Table 2.

These differences in persistence further indicate that the focus of risk management for low‐degradation systems should progressively escalate as material persistence increases: from restricting application scenarios that generate systemic exposure, to continuous post‐application surveillance, and further upstream to pre‐application traceability design. For HNTs, poorly soluble aluminosilicates generally exhibit low gastrointestinal absorption and low systemic bioavailability; when in vivo application is necessary, scenarios such as the gastrointestinal tract that can limit systemic exposure may therefore be prioritized (Priest 1993).

For AAOs and TiO_2_ nanotubular arrays, the risks of surface wear, structural damage, and particle detachment should be thoroughly evaluated before application, followed by long‐term surveillance using repeatable quantitative indicators. Patient education should also support timely recognition of relevant abnormalities and prompt reassessment (Crisponi et al. 2013; Luo et al. 2020). Because the boron nitride framework of BNNTs is the most stable and lacks clearly established in vivo degradation and metabolic pathways (Merlo et al. 2018), their safety management should additionally include reliable signal labeling and long‐term tracking strategies. For localized and traceable systems, abnormal migration or tissue reactions should prompt further assessment of the necessity and feasibility of intervention or removal.

These factors and their associated mechanisms are interrelated, and their effects depend on the specific material system, exposure conditions, and functional requirements. No universal parameter hierarchy or optimal combination therefore applies to all nanotube systems. The correspondences presented in Table 3 should not be used to establish direct causality between a single factor and a pathological outcome, but they may provide a reference for subsequent experimental validation, adjustment of design strategies, and identification of risk‐management priorities.

## 5 Biomedical Applications of Nanotubes

Owing to their unique structural characteristics and exceptional physicochemical properties, nanotubes have demonstrated tremendous potential in the biomedical field (Ahmad et al. 2023). In disease therapy, their hollow tubular architecture allows efficient loading of drugs, genes, and other bioactive molecules, enabling targeted delivery and controlled release, thereby enhancing therapeutic efficacy while minimizing systemic toxicity (Das et al. 2025). In disease diagnosis, nanotubes can serve as high‐performance biosensing platforms, capable of precisely capturing biomarkers for early and rapid disease detection (El Zanin et al. 2025; Bhabal et al. 2023; Harrison and Atala 2007). In addition, their distinct physical properties can promote cellular adhesion, proliferation, and differentiation and, in some systems, inhibit tumor growth. In tissue engineering, they provide ideal scaffolds for cell growth, tissue repair, and regeneration (Amiryaghoubi et al. 2021).

Ocular drug delivery represents a specialized application scenario in which nanotube‐based controlled‐release and targeting designs may be further extended. Conventional ocular formulations commonly exhibit short local residence times, low bioavailability, and limited delivery to the posterior segment because of tear‐film clearance, the corneal barrier, and the blood–ocular barriers. Studies of bioinspired nanocarriers indicate that sustained release, enhanced tissue retention, and receptor‐mediated targeting may improve ocular delivery; material accumulation, intraocular inflammation, and local cytotoxicity must nevertheless be evaluated in parallel (Chen et al. 2023).

### 5.1 Carbon Nanotubes (CNTs)

Carbon nanotubes consist of sp^2^‐hybridized carbon atoms arranged as graphitic coaxial walls around a hollow lumen. Their high surface area, rigidity, and electrical and thermal conductivity support three principal biomedical functions: molecular loading, optical/electrical signal transduction, and mechanical reinforcement of tissue‐engineering scaffolds (Liu et al. 2024; Lohani et al. 2025).

#### 5.1.1 Drug Loading and Controlled Release of CNTs

In drug‐delivery systems, the CNT surface and lumen provide high‐capacity loading sites, while introduced functional groups serve as attachment points for therapeutic molecules and release‐controlling linkers. Targeting ligands can further direct functionalized CNTs toward pathological sites, improving therapeutic selectivity and reducing off‐target toxicity (Das et al. 2025; Bhabal et al. 2023).

Thakur et al. reviewed MWCNT functionalization strategies using folic acid and tumor‐targeting antibodies to deliver doxorubicin and paclitaxel. These systems preferentially bound receptor‐overexpressing tumor cells, improving antitumour efficacy while reducing toxicity to healthy tissues (Thakur et al. 2024).

#### 5.1.2 Imaging and Diagnostic Applications of CNTs

Benefiting from their unique optical and electrical properties, CNTs have emerged as promising contrast agents and probes in bioimaging. In photoacoustic imaging, CNTs absorb near‐infrared light and efficiently convert thermal energy into acoustic signals. Their excellent electrical conductivity and large surface area amplify signal responses, allowing ultrasensitive detection. Combined with high carrier mobility, CNT‐based platforms enable rapid detection of analyses at low detection limits, particularly suitable for cancer biomarker monitoring and early cancer diagnosis (Lohani et al. 2025; El Zanin et al. 2025; Acharya et al. 2024; Gao et al. 2024).

Aydın et al. fabricated a biosensor by electrodepositing SWCNTs onto an indium tin oxide electrode followed by polymerization of oxetane‐2‐methyl 3‐(1H‐pyrrol‐1‐yl) propionate. The resulting biosensor exhibited high selectivity for calreticulin (CALR) over other biomarkers, including neuron‐specific enolase (NSE), interleukin‐1α (IL‐1α), interleukin‐1β (IL‐1β), TNF‐α, and interleukin‐6 (IL‐6). Under optimal conditions, the sensor showed a linear response in the range of 0.015–60 pg/mL, an extremely low detection limit (4.6 fg/mL), and a sensitivity of 0.43 kΩ·pg.^−1^·mL·cm^−2^. In human serum samples, CALR recovery rates ranged from 95.28% to 108.30%, and 61.69% of the initial signal was retained after 6 weeks at 4°C, demonstrating excellent stability (Aydın et al. 2022).

**FIGURE 1:**
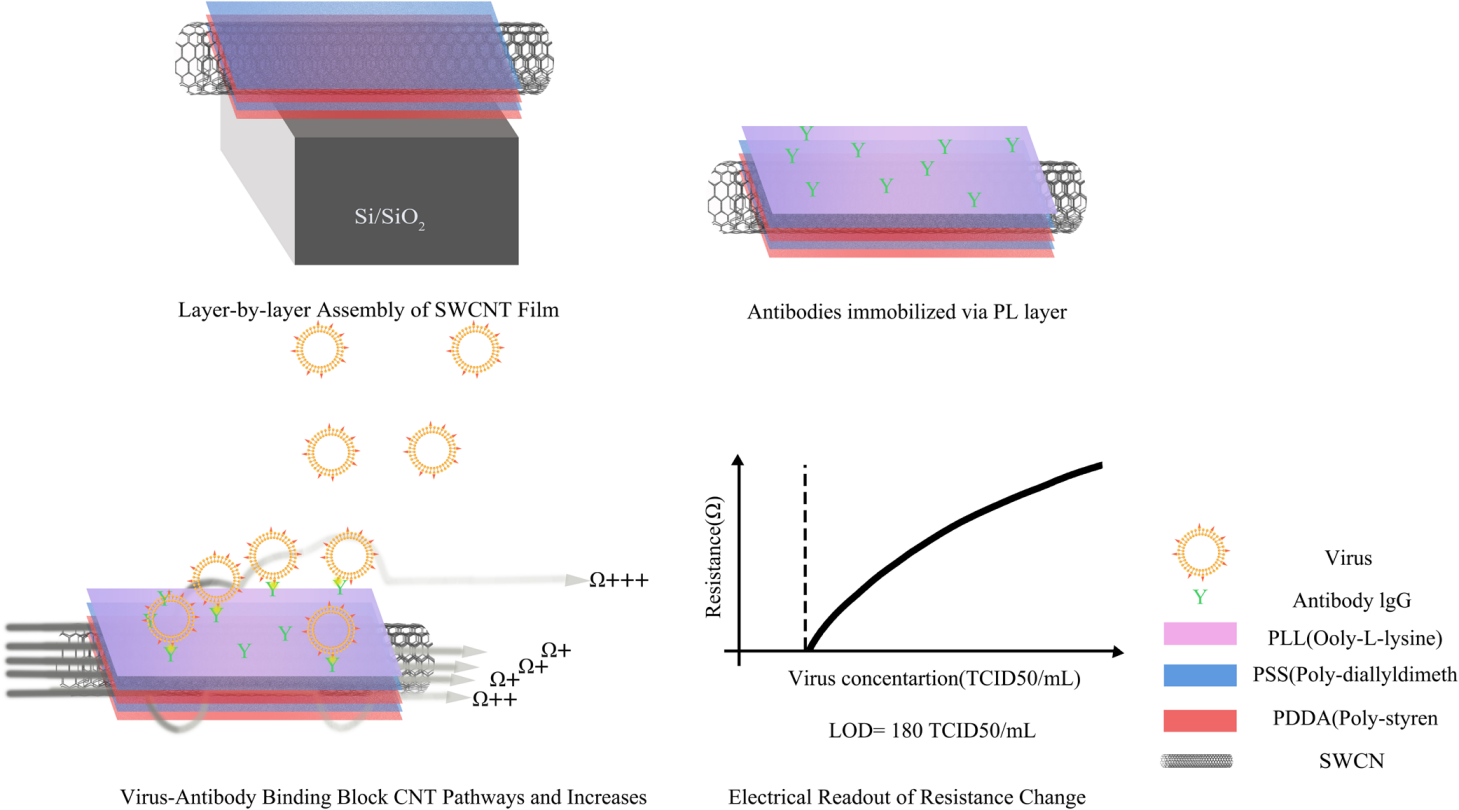
Schematic illustration of a SWCNT‐based immunosensor for virus detection via layer‐by‐layer assembly and antibody‐mediated signal transduction.

Lee et al. constructed a layer‐by‐layer self‐assembled sensing film by alternately depositing carboxylated SWCNTs and polyelectrolytes. Formation of antibody–virus complexes altered the electrical resistance of the film, enabling detection of simian immunodeficiency virus. Target‐virus binding induced a measurable increase in resistance, with a detection limit of 180 median tissue culture infectious dose units per milliliter (TCID_50_/mL). The sensor exhibited good selectivity against non‐target viruses, a background signal below 12%, and high reproducibility and stability (Lee et al. 2010) (Figure 1).

Furthermore, CNTs can be integrated with graphene to achieve synergistic electrical and mechanical performance. Wang et al. combined 5 wt% graphene with SWCNTs via ultrasonication to form a homogeneous suspension, which was drop‐cast onto a polyurethane (PU) substrate and dried to produce a SWCNT/graphene‐PU (SCG/PU) electronic skin (SCGe‐skin). The SCGe‐skin exhibited gauge factors of 964 at 0%–30% strain and 2743 at 30%–60% strain, with a detection limit as low as 0.5% strain, demonstrating exceptional mechanosensing performance (Wang et al. 2023).

#### 5.1.3 Tissue Engineering and Regenerative Repair of CNTs

Due to their graphitic coaxial tubular structure, individual CNTs exhibit an extraordinarily high Young's modulus of approximately 1.0–1.8 TPa, nearly 50 times greater than that of steel, and a tensile strength of 50–60 GPa, far exceeding most engineering materials (Assali et al. 2022). Taking advantage of this outstanding mechanical performance, CNTs can be incorporated as nano‐reinforcing phases into various biomedical matrices, markedly improving the mechanical strength of devices and tissue‐engineering scaffolds.

Maksym et al. fabricated hydroxyapatite (HA)/MWCNT composites by mixing low‐defect MWCNTs, synthesized via chemical vapor deposition, with HA, followed by vacuum sintering at 1100°C for 1 h. The composite containing 0.5 wt% MWCNTs exhibited the highest hardness and compressive strength (5.7 GPa) among all tested samples (Barabashko et al. 2022).

In bone tissue engineering, Jing et al. prepared oxidized MWCNT/collagen/hydroxyapatite scaffolds, where incorporation of MWCNTs significantly enhanced the compressive modulus. When rat bone marrow mesenchymal stem cells were seeded onto the scaffolds, those containing 0.5 wt% CNTs showed markedly enhanced cell proliferation and osteogenic differentiation, with significantly upregulated bone sialoprotein (BSP) and osteocalcin (OCN) gene and protein expression compared with pure collagen/hydroxyapatite scaffolds. In vivo experiments using cranial defect models in adult female Sprague–Dawley (SD) rats, the 0.5 wt% CNT scaffold group exhibited greater new bone formation and higher BSP/OCN expression than the control group (Jing, Wu, et al. 2017).

### 5.2 Boron Nitride Nanotubes (BNNTs)

BNNTs possess a distinctive set of physicochemical characteristics. Their hexagonal boron‐nitride lattice provides high chemical stability, good biocompatibility, and intrinsic electrical insulation, while their hollow tubular morphology supports molecular encapsulation. BNNTs also exhibit piezoelectric responsiveness, neutron‐capture capability arising from boron atoms, and tuneable antibacterial activity through surface coating or polymer wrapping (Kodali et al. 2022). These properties make them suitable for magnetic resonance imaging (MRI) contrast enhancement, mechanically or ultrasonically driven localized electrical stimulation, bone and neural regeneration, and boron neutron capture therapy.

#### 5.2.1 Imaging Enhancement of BNNTs

Calucci et al. loaded iron nanoparticles into BNNTs and evaluated the longitudinal (*r*_1_) and transverse (*r*_2_) proton relaxivities of the Fe‐BNNT/poly(L‐lysine) aqueous dispersions at different magnetic field strengths. The results showed that both *r*_1_ and *r*_2_ relaxivities increased at 1.5 T and 7.05 T, while the *r*_2_ value at 1.5 T exceeded that of commercial superparamagnetic iron oxide nanoparticles and increased further at the higher magnetic field (Calucci et al. 2010).

Similarly, when gadolinium ions (Gd^3+^) were encapsulated into BNNTs to form Gd‐BNNT composites, Ciofani et al. demonstrated that these nanostructures significantly enhanced MRI contrast at both 3 T and 7 T, highlighting their potential as multifunctional imaging–therapeutic nanoplatforms (Menichetti et al. 2011; Ciofani, Boni, et al. 2013).

#### 5.2.2 Tissue Engineering and Regenerative Repair of BNNTs

The piezoelectric behavior of BNNTs is one of their most remarkable physical characteristics. Kang et al. demonstrated that the electroactive strain of 2 wt% BNNT/polyimide composites arises from the superposition of linear piezoelectric and nonlinear electrostrictive components. After mechanical stretching, the piezoelectric coefficient (*d*_33_) increased from 0.84 to 4.71 pm V^−1^, while the electrostrictive coefficient (*M*_33_) increased from 3.07 × 10^−19^ to 1.79 × 10^−18^ m^2^ V^−2^ (Kang et al. 2015).

Exploiting this property, Ciofani et al. investigated the piezoelectric stimulation effect of BNNTs on cells. Under ultrasound irradiation, neuron‐like PC12 cells were co‐cultured with BNNTs, where the nanotubes functioned as nanotransducers, polarizing and converting mechanical ultrasound energy into localized electrical stimuli. After 9 days of stimulation, the BNNT‐treated PC12 cultures exhibited a 30% increase in neurite outgrowth compared with control cells (Ciofani et al. 2010).

**FIGURE 2:**
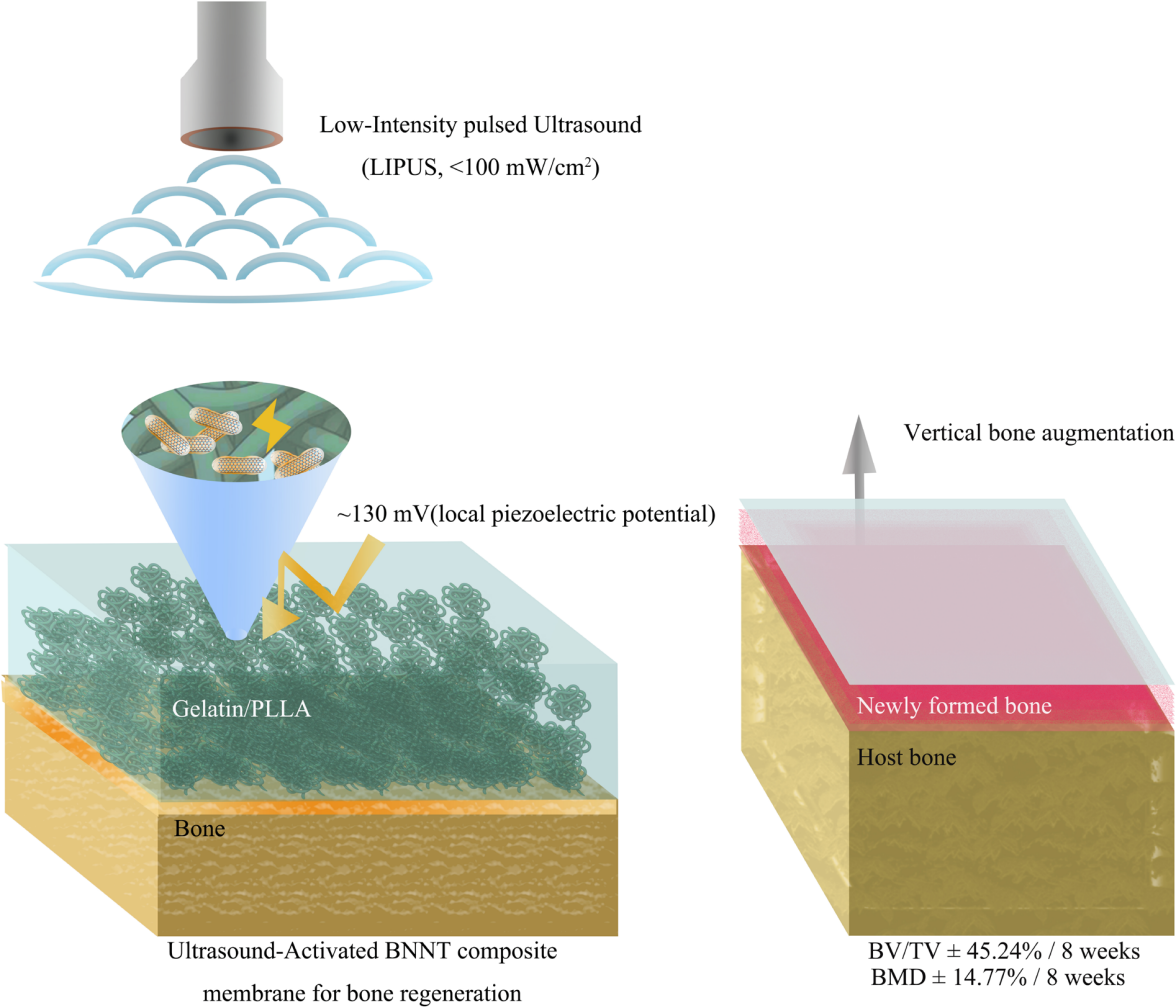
Piezoelectric BNNT composite membranes enable ultrasound‐assisted vertical bone augmentation.

In bone tissue engineering, conventional guided bone regeneration membranes lack both osteoinductivity and antibacterial activity. Zhu et al. fabricated an electrospun poly(L‐lactic acid) (PLLA)/gelatin/polydopamine (PDA)‐BNNT composite membrane containing dispersed BNNTs. At an ultrasound intensity below 100 mW/cm^2^, the membrane generated a piezoelectric voltage of approximately 130 mV and effectively promoted osteogenesis in vivo, demonstrating potential for the repair of vertical bone defects (Zhu et al. 2025) (Figure 2).

#### 5.2.3 Antibacterial Activity and Boron Neutron Capture Therapy of BNNTs

Nithya et al. synthesized polyethyleneimine‐coated BNNTs (PEI‐BNNTs) and Pluronic F127‐wrapped BNNTs (F‐BNNTs), which were subsequently loaded with tamoxifen and paclitaxel, respectively. Pristine BNNTs showed no evident antibacterial activity, whereas PEI‐BNNTs strongly inhibited *Escherichia coli* and *Staphylococcus aureus* . Drug‐loaded F‐BNNTs inhibited cancer‐cell proliferation and induced apoptosis even at low concentrations (Nithya and Pandurangan 2014).

Díez‐Pascual et al. coated BNNTs with PEG and embedded them in poly(propylene fumarate) (PPF) matrices to create BNNT‐PEG/PPF composites. These composites exhibited lower friction coefficients and wear rates and dose‐dependent bactericidal activity against *Staphylococcus aureus* and *E. coli* , while showing no evident cytotoxicity toward human dermal fibroblasts. Their stiffness and strength under physiological conditions were also greater than those of pristine PPF, supporting their use in bone‐tissue regeneration (Díez‐Pascual and Díez‐Vicente 2016).

Because boron nuclei can capture thermal neutrons and subsequently emit highly cytotoxic α‐particles, BNNTs can serve as targeted boron carriers for boron neutron capture therapy (BNCT). By conjugating tumor‐recognizing ligands onto BNNT surfaces, they can be directed to tumor sites, where subsequent neutron irradiation generates α‐particles to selectively destroy cancer cells (Bae et al. 2024).

Ciofani et al. first employed poly‐L‐lysine (PLL) as a dispersant to prepare PLL‐coated BNNTs (PLL‐BNNTs) through noncovalent coating, achieving stable aqueous dispersions. They then conjugated folic acid (FA) to obtain FA‐functionalized PLL‐BNNTs (FA‐PLL‐BNNTs). In vitro studies demonstrated preferential uptake of FA‐PLL‐BNNTs by glioblastoma (T98G) cells, with minimal internalization by normal human fibroblasts (Ciofani et al. 2009).

Yamana et al. further conjugated human epidermal growth factor receptor 2 (HER2)‐targeting antibodies to a BNNT/β‐glucan complex, forming immunoglobulin G (IgG)‐functionalized BNNT/β‐glucan nanocomposites. In SK‐OV‐3 xenograft mice, intravenous administration of this complex led to boron accumulation peaking at 36.8 μg per 100 mg tumor tissue within 24 h, exhibiting remarkable tumor selectivity (tumor‐to‐blood ratio [T/B] = 390; tumor‐to‐normal‐tissue ratio [T/N] = 170). The anticancer efficacy was approximately 30 times higher than that of the conventional BNNTs/L‐boronophenylalanine/fructose formulation (Yamana et al. 2023).

### 5.3 Titanium Dioxide Nanotubes (TiNTs)

TiNTs generated via anodic oxidation of titanium substrates form ordered tubular arrays with excellent biocompatibility and inherent osteophilicity. Their hollow channels act as localized drug reservoirs, enabling sustained and stimuli‐responsive release at implant interfaces. In addition, TiNT coatings promote strong osseointegration by enhancing osteoblast adhesion, proliferation, and mineralization, thereby improving the mechanical stability of implants. When further functionalized with antimicrobial agents or nanostructured surface layers, TiNTs provide long‐term antibacterial activity. Thus, the key biomedical utility of TiNTs lies in controlled drug release, scaffold‐bone interface reinforcement, and antibacterial surface engineering.

#### 5.3.1 Drug Delivery by TiNTs

Similar to CNTs and BNNTs, the hollow structure of TiNTs can function as a micro‐reservoir for localized drug delivery, particularly when integrated with implant surfaces. Păun et al. fabricated Y‐branched TiNT arrays and loaded them with tetracycline. Compared with uncoated controls, the arrays achieved approximately 50% higher antibacterial efficiency against *E. coli* and *S. aureus* without altering the structural or physicochemical stability of the TiNT coating (Păun et al. 2023).

**FIGURE 3:**
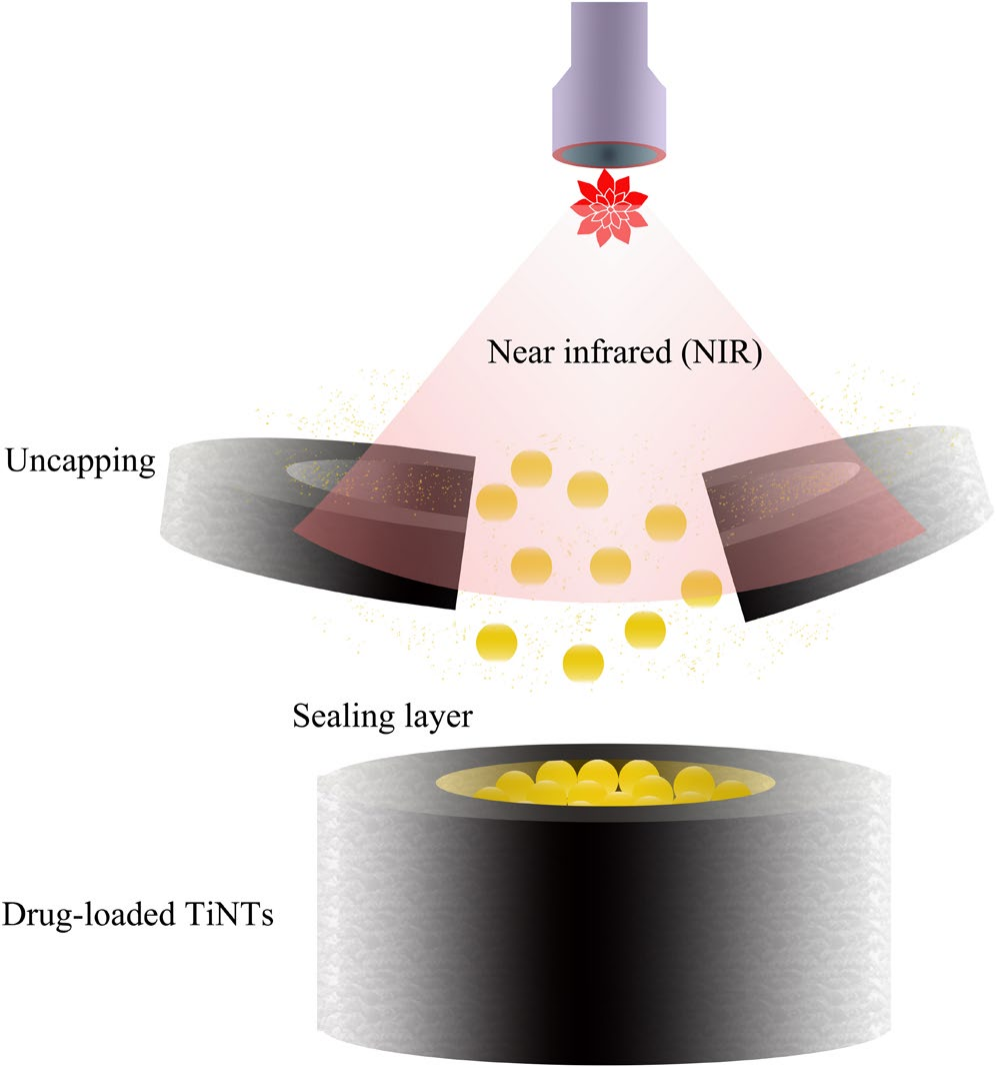
Stimuli‐responsive uncapping strategy for controlled drug release from nanotubular reservoirs.

Furthermore, by controlling the aspect ratio (length‐to‐diameter) of TiNTs, both the drug loading capacity and release kinetics can be finely tuned (Li et al. 2021). Incorporation of thermo‐ or photo‐responsive sealing layers further enables stimuli‐triggered release, such as near‐infrared‐induced uncapping, providing precise spatiotemporal control over local drug administration (Figure 3).

#### 5.3.2 Tissue Engineering and Regenerative Repair of TiNTs

As a ceramic nanostructure, TiNTs exhibit a high elastic modulus and hardness, as well as remarkable osteophilicity, making them excellent candidates for bone‐implant interfaces. Implants featuring TiNT arrays have demonstrated outstanding mechanical reinforcement and osseointegration performance.

For instance, Bjursten et al. simultaneously implanted TiNT arrays and sandblasted TiO_2_ surfaces into rabbit tibiae. After 4 weeks, mechanical pull‐out testing revealed that the bone‐implant interfacial strength of TiNT‐coated implants was nearly nine times higher than that of the sandblasted TiO_2_ controls. Histological analyzes confirmed that TiNT‐coated implants exhibited a larger bone‐implant contact area, greater new bone formation, and higher calcium and phosphorus deposition on the surface (Bjursten et al. 2010).

Parcharoen et al. prepared hydroxyapatite‐coated TiNT arrays (HA‐TiNTs) by electrodeposition and evaluated the adhesion and proliferation of MC3T3‐E1 pre‐osteoblasts. After 3 days of culture, cell proliferation on HA‐TiNTs reached 117%, substantially higher than the 59% observed on pure titanium. Mechanical pull‐off testing showed an adhesion strength of up to 21 MPa between the HA‐TiNT coating and the substrate, indicating good coating stability (Parcharoen et al. 2016; Chang et al. 2024).

#### 5.3.3 Antibacterial Activity and Biointerface Modulation of TiNTs

TiO_2_ nanotube coatings can be engineered to endow implant surfaces with long‐lasting antibacterial properties. TiNT arrays fabricated by anodization combined with ion implantation and loaded with Ag^+^, for example, inhibited pathogenic bacterial growth over extended periods while exhibiting low cytotoxicity toward human cells (Yao et al. 2025). Spiderweb‐like TiNT networks can further enhance antibacterial activity by increasing the contact interface between silver nanoparticles and bacterial membranes (Chokesawatanakit et al. 2021). Alternatively, graphene layers on TiNT surfaces can produce a “nanoknife effect”, in which sharp graphene edges mechanically disrupt bacterial membranes (Rahnamaee et al. 2022).

### 5.4 Halloysite Nanotubes (HNTs)

Halloysite nanotubes are naturally occurring aluminosilicate nanotubes with a hollow tubular morphology, high aspect ratio, and chemically distinct inner and outer surfaces (Ganapathy et al. 2022). Their positively charged alumina lumen and negatively charged siloxane exterior support selective drug encapsulation and flexible surface functionalization, making HNTs particularly suitable for controlled‐release systems (Massaro et al. 2018). HNTs also exhibit good biocompatibility and moderate mechanical strength and can be uniformly dispersed in natural or synthetic polymer matrices, allowing them to serve as reinforcing components in composite biomaterials. Their natural abundance, low cost, and tunable structure–property relationships have supported applications mainly in sustained drug release and tissue‐engineering‐based regenerative repair.

#### 5.4.1 Drug Loading and Controlled Release of HNTs

Massaro et al. functionalized HNT surfaces with cyclodextrin (CD) via microwave‐assisted synthesis and subsequently loaded the antifungal drug clotrimazole. The CD‐functionalized HNTs exhibited a drug loading capacity of 5.4 wt% and an encapsulation efficiency of 80%, markedly higher than pristine HNTs (1.5 wt%, 47%). In phosphate‐buffered saline (pH 7.4), the CD‐HNTs released ~60% of clotrimazole over 150 min, whereas unmodified HNTs released nearly all drug within 30 min, demonstrating the sustained‐release behavior of the functionalized system (Massaro et al. 2018).

Yang et al. synthesized chitosan oligosaccharide (COS)‐grafted HNTs (HNTs‐g‐COS) as doxorubicin (DOX) carriers for breast cancer therapy. The half‐maximal inhibitory concentration (IC_50_) of DOX‐loaded HNTs‐g‐COS against MCF‐7 cells was 1.17 μg/mL, lower than the 2.43 μg/mL observed for free DOX. In 4T1 tumor‐bearing mice, the tumor‐inhibition rate reached 83.5%, compared with 46.1% for free DOX. No substantial systemic toxicity was detected, and myocardial damage was minimal, indicating both enhanced efficacy and improved biosafety (Yang et al. 2016).

#### 5.4.2 Tissue Engineering and Regenerative Repair of HNTs

In addition to their abundant surface hydroxyl groups and high specific surface area, HNTs exhibit a Young's modulus of 130–140 GPa and a tensile strength of 10.8 MPa. Combined with their low density, these properties allow HNTs to be uniformly dispersed in natural biopolymers such as chitosan, sodium alginate, and hyaluronic acid, as well as in synthetic polymers like PEG, poly(vinyl alcohol), and poly(acrylic acid). The resulting composites display enhanced mechanical strength and improved nutrient absorption capacity of the engineered tissues (Chiew et al. 2014; Ghadirian et al. 2024).

**FIGURE 4:**
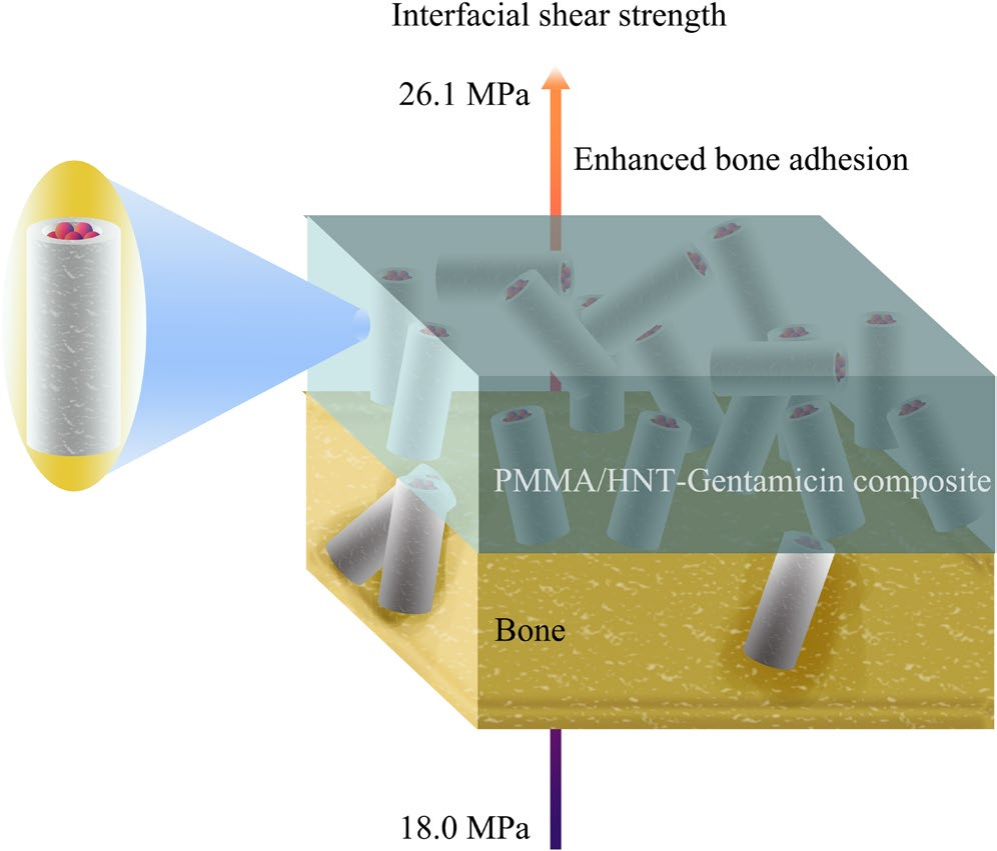
Enhanced antibacterial performance and bone adhesion of PMMA/HNT‐gentamicin composite coatings.

In bone‐tissue repair, HNTs have been incorporated into both bone cements and hydrogel scaffolds. Wei et al. first loaded gentamicin sulfate onto HNTs by ultrasonication and then incorporated the drug‐loaded HNTs into poly(methyl methacrylate) (PMMA) to produce PMMA/HNT/gentamicin composites. Pristine HNTs released 94% of gentamicin within 48 h, whereas the composite prolonged release to 300–400 h. Incorporation of 5 wt% HNTs also increased tensile strength from 18.0 to 26.1 MPa and substantially enhanced bone adhesion (Wei et al. 2012) (Figure 4).

Huang et al. constructed HNT/gelatin methacryloyl (GelMA) composite hydrogels by photopolymerization. Increasing HNT content substantially increased the compressive modulus, which reached approximately 0.4 MPa in the 7 wt% HNT group. While maintaining good compatibility with human dental pulp stem cells, the system upregulated osteogenesis‐related genes and proteins, including collagen type I (COL1), runt‐related transcription factor 2 (RUNX2), bone morphogenetic protein 4 (BMP4), and bone sialoprotein (BSP). In a rat calvarial‐defect model, hydrogels containing 5% and 7% HNTs generated more mineralized bone tissue and demonstrated favorable in vivo bone‐regeneration capacity (Huang et al. 2019).

Building on this approach, Ou et al. introduced nanosilver (nAg) into HNT/GelMA hydrogels to construct nAg/HNT/GelMA hybrid scaffolds. The system showed good compatibility with both human periodontal ligament stem cells and macrophages, suppressed the release of multiple pro‐inflammatory cytokines, and increased anti‐inflammatory factors, thereby reducing the adverse influence of an inflammatory environment on osteogenic differentiation. Nanosilver simultaneously conferred broad‐spectrum antibacterial activity against Gram‐positive and Gram‐negative bacteria. In an infected rat calvarial‐defect model, the scaffold combined infection control, regulation of the osteoimmune microenvironment, and promotion of new‐bone formation, demonstrating that HNT‐based hybrid scaffolds can integrate mechanical reinforcement, antibacterial activity, and immunomodulation within a single bone‐repair platform (Ou et al. 2020).

In skin regeneration, Sandri et al. dispersed HNT powder in a 1% chitosan‐oligosaccharide solution and freeze‐dried the mixture to produce a composite. In vitro, the composite promoted fibroblast proliferation more effectively than either HNTs or chitosan oligosaccharide alone. In a rat‐burn model, early re‐epithelialization, hemostasis, and angiogenesis were observed by day 7; by day 18, skin architecture was largely restored, and hair‐follicle regeneration was evident (Sandri et al. 2017).

### 5.5 Anodic Aluminium Oxide Nanotubes (AAOs)

AAOs exhibit a highly ordered nanoporous architecture formed through electrochemical anodization, providing precise control over pore diameter, channel length, surface area, and pore density (Sandri et al. 2017; Kapruwan et al. 2020). These structural parameters directly determine their biomedical performance. The vertically aligned nanochannels serve as well‐defined reservoirs for therapeutic molecules, enabling sustained, diffusion‐mediated, and geometry‐tunable drug release. Their high chemical stability, surface hydroxyl groups, and excellent biocompatibility allow AAOs to immobilize nucleic acids, enhance biosensing signal amplification, and sustain antibacterial activity when loaded with antimicrobial ions or functional coatings. Moreover, the nanoscale topography of AAO membranes can modulate cell adhesion, morphology, and differentiation, making them effective templates for tissue interface engineering. Taken together, the structural tunability, chemical robustness, and favorable biointerface properties of AAOs naturally align with their major biomedical applications, including controlled drug release, diagnostic sensing, antibacterial surface engineering, and tissue regeneration.

#### 5.5.1 Drug Loading and Antibacterial Activity of AAOs

Kapruwan et al. fabricated AAO nanoporous structures as intraosseous implants for drug delivery. Their results revealed that the AAO framework enabled sustained and slow drug release within ex vivo bone tissue models. By adjusting the pore diameter and tube length of AAOs, the release duration could be significantly extended, producing a smoother and more controlled release profile (Sandri et al. 2017).

**FIGURE 5:**
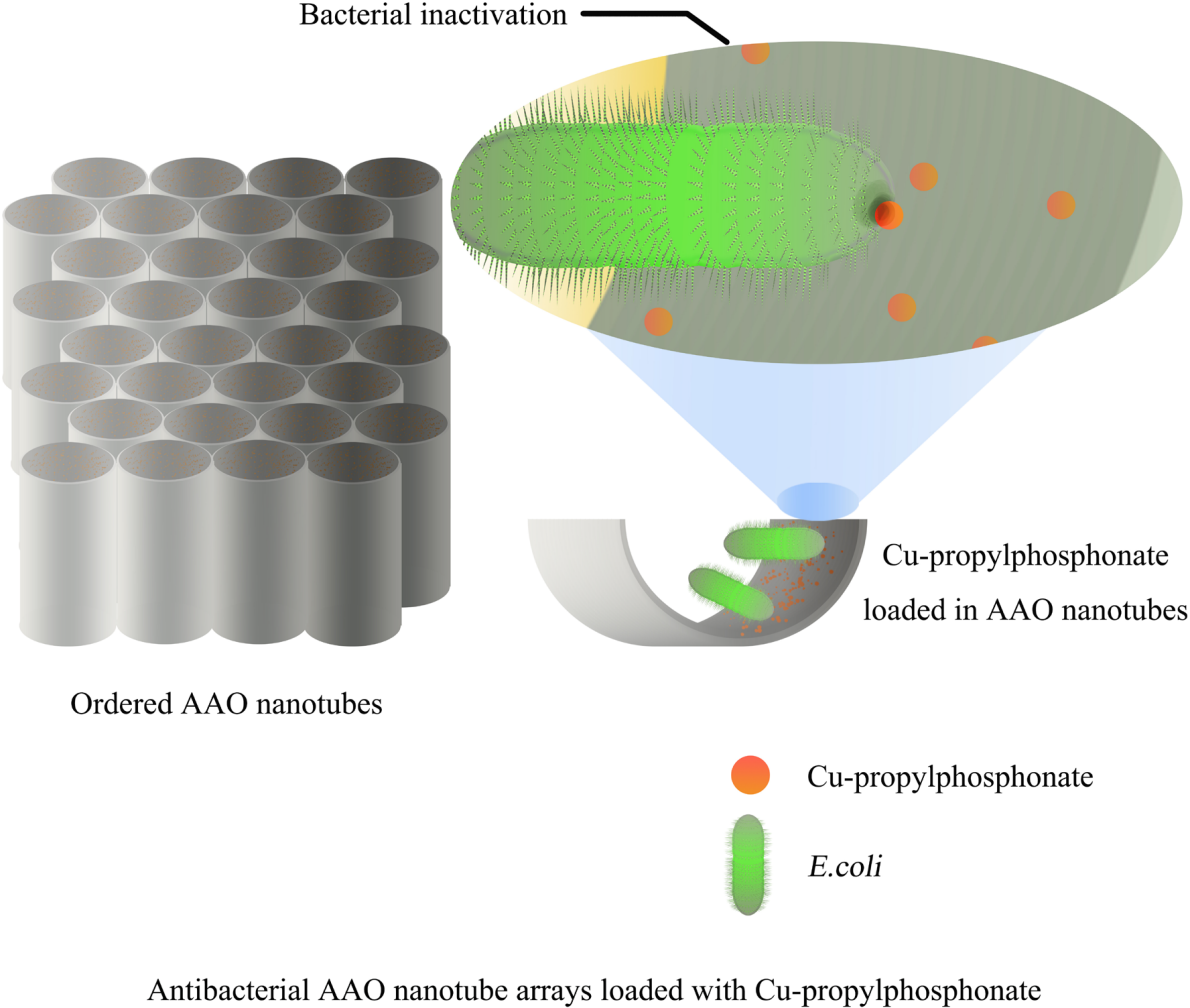
Antibacterial AAO nanotube arrays loaded with Cu‐propylphosphonate.

Schabikowski et al. loaded copper propylphosphonate complexes into AAO nanotubes and evaluated their antibacterial efficacy against several strains of *E. coli* (K12, R2, R3, R4). The AAO‐Cu composite exhibited strong antibacterial activity (Kapruwan et al. 2020), surpassing that of ciprofloxacin, bleomycin, and cloxacillin, indicating its potential as an effective antimicrobial surface coating (Figure 5).

#### 5.5.2 Imaging and Diagnostic Applications of AAOs

Makela et al. developed a severe acute respiratory syndrome coronavirus 2 (SARS‐CoV‐2) nucleic‐acid detection platform by immobilizing deoxyribonucleic acid (DNA) duplexes and DNA–ribonucleic acid (RNA) hybrids within AAO nanopores. The resulting biosensor achieved a signal sensitivity two orders of magnitude higher than that of planar glass substrates, enabling high‐throughput and rapid genetic detection (Schabikowski et al. 2022).

#### 5.5.3 Tissue Engineering and Regenerative Repair of AAOs

In vitro studies have demonstrated that AAO membranes can promote cell adhesion, proliferation, and differentiation. Popat et al. cultured mouse bone marrow stromal cells on nanoporous AAO and amorphous alumina (control) surfaces. Over a 3‐week period, the nanoporous AAO surface enhanced cell adhesion and proliferation, increased cell viability by ~45%, alkaline phosphatase activity by 35%, and matrix production by 50% compared to the amorphous control (Makela et al. 2021).

However, the pore diameter of AAO membranes may affect cell morphology and inflammatory response in vivo. Shiuli Pujari‐Palmer et al. cultured W20‐17 bone marrow stromal cells on AAO membranes with pore sizes of 20, 100, and 200 nm. Cells on 20 nm pores were mostly round, whereas those on 200 nm pores exhibited elongated morphologies (Popat et al. 2007). In a related study, Pujari et al. implanted 20 and 200 nm AAO membranes subcutaneously in mice and observed fibroblast and macrophage behavior. The 200 nm‐pore membranes induced a stronger inflammatory response than 20 nm pores, highlighting the importance of pore‐size‐dependent biocompatibility (Pujari‐Palmer et al. 2014).

### 5.6 Lipid Nanotubes (LNTs)

LNTs possess a distinctive bilayer tubular architecture. Their hollow lumen, hydrophobic bilayer membrane, and hydrophilic exterior enable simultaneous incorporation of hydrophobic and hydrophilic drugs, supporting combination therapy and controlled release (Pujari et al. 2014; Bi et al. 2022). The bilayer membrane also enhances mechanical stability and reduces premature leakage during circulation. Their size, aspect ratio, and membrane composition influence passive biodistribution and circulation behavior in vivo, while their surfaces can be further conjugated with targeting ligands such as peptides, antibodies, or nucleic‐acid aptamers to enable receptor‐mediated uptake and improve therapeutic selectivity.

#### 5.6.1 Drug Loading and Controlled Release of LNTs

**FIGURE 6:**
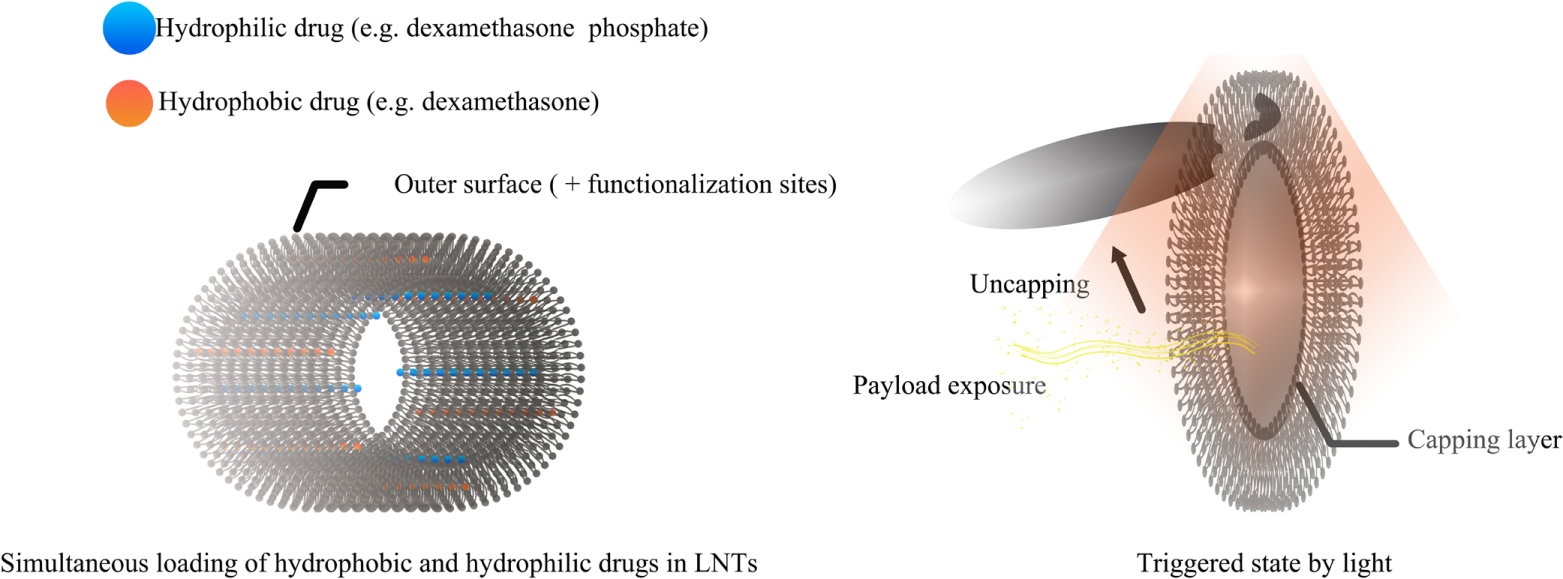
Multimodal drug loading and stimuli‐responsive release in lipid nanotubes.

LNTs have been shown to be an unusual drug carrier, as the bilayer structure of LNTs allows hydrophobic drugs to be embedded within the membrane, while the inner lumen and outer surface provide additional sites for hydrophilic drug loading. For instance, self‐assembled 1,2‐bis(tricosa‐10,12‐diynoyl)‐sn‐glycero‐3‐phosphocholine LNTs can simultaneously encapsulate hydrophobic dexamethasone and hydrophilic dexamethasone phosphate (Wang et al. 2022), enabling combination therapy using a single carrier (Figure 6).

Furthermore, the release profile can be modulated through stimuli‐responsive strategies, such as pH‐, enzyme‐, or light‐triggered uncapping, enabling precise spatiotemporal control of therapeutic action (Wang et al. 2020).

A similar thermoresponsive‐release principle has been validated in non‐tubular lipid carriers. The paclitaxel thermosensitive liposomes constructed by Huang et al. exhibited a phase‐transition temperature of approximately 42.6°C, remained relatively stable below that temperature, and released the drug much more rapidly at 43°C–45°C. When combined with gold‐nanostar‐mediated local heating, the system also increased tumor‐tissue permeability and drug retention and enhanced treatment of drug‐resistant tumors. Although this system was not an LNT, it provides a mechanistic reference for designing thermoresponsive LNT release systems based on lipid‐bilayer phase transitions (Huang et al. 2022).

#### 5.6.2 Targeted Delivery Strategies of LNTs

LNTs can achieve targeting through both passive and active mechanisms. In passive targeting, LNT composition, size, and aspect ratio influence circulation behavior and biodistribution in vivo (Lin et al. 2023). In active targeting, functional ligands including peptides, antibodies, and nucleic‐acid aptamers can be conjugated to LNT surfaces to enable receptor‐mediated uptake by specific tissues or cell populations (Cheng et al. 2025; van Alem et al. 2021). The two mechanisms can act synergistically to improve therapeutic selectivity and reduce off‐target toxicity.

### 5.7 Polymeric Nanotubes (PNTs)

PNTs combine highly tuneable molecular architecture, intrinsic biocompatibility, and programmable degradation. Polymer composition, chain arrangement, and crosslinking chemistry can be adjusted to precisely control lumen size, surface functionality, mechanical strength, and degradation kinetics. PEG‐based polymeric nanotubes reported by Alghamdi et al. for example, exhibited tuneable drug loading and sustained‐release profiles (Alghamdi et al. 2020). The internal cavity and modifiable surface can accommodate therapeutic molecules and functional components, while further coating with biodegradable polymers such as PLGA can regulate release kinetics and cytocompatibility (Davoodian et al. 2020). In tissue engineering, designable mechanical properties and biomimetic tubular scaffold architectures can promote cell adhesion, proliferation, and lineage‐specific differentiation.

#### 5.7.1 Drug Loading and Controlled Release of PNTs

The application of PNTs in drug delivery takes advantage of their large specific surface area and tubular geometry, which allows for high drug loading capacities and controlled release behaviors (Kerr et al. 2022). For example, the drug loading efficiency of PEG nanotubes can be adjusted by varying the degree of carboxylic functionalization (Tyler et al. 2016). Similarly, PLGA‐based nanotubular shells can be engineered to provide sustained release profiles and improved cell compatibility (Heo et al. 2025).

Shen et al. developed PEG–PLA–PEG triblock copolymer nanotubes capable of co‐loading hydrophilic cisplatin and hydrophobic paclitaxel, demonstrating dual‐drug delivery from a single carrier (Shen et al. 2019). Compared with spherical nanocarriers of similar size, tubular polymeric nanocarriers exhibit higher cellular uptake and deeper tissue penetration, attributed to their anisotropic morphology (Abidian et al. 2006). Furthermore, variations in crosslinking chemistry can significantly influence the loading capacity and release kinetics of PNTs (Kerr et al. 2022).

#### 5.7.2 Imaging and Diagnostic Applications of PNTs

In biomedical imaging and diagnostic applications, PNTs serve as nanoprobes or contrast‐agent carriers, providing distinctive advantages for multimodal imaging. Their internal cavities and external surfaces can simultaneously encapsulate contrast agents, fluorescent dyes, or radioisotopes, enabling integrated imaging modalities with improved resolution and sensitivity (Xiao et al. 2023).

#### 5.7.3 Tissue Engineering and Regenerative Repair of PNTs

The mechanical performance of polymers such as PLA, PLGA, and PCL can be adjusted through molecular design, making them ideal as nanotubular scaffolds in tissue engineering (Kerr et al. 2022; Abidian et al. 2006). PEG can be incorporated as a structural additive to enhance the flexibility and hydrophilicity of the scaffold, resulting in three‐dimensional (3D) hybrid constructs suitable for soft‐tissue regeneration (Sang et al. 2022).

**FIGURE 7:**
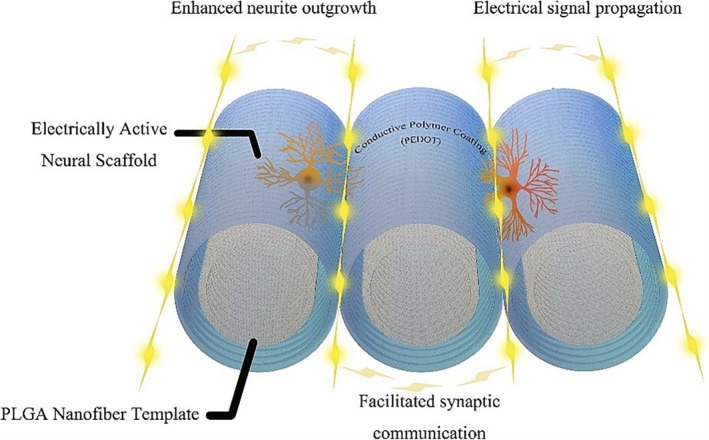
PEDOT‐based conductive nanotube scaffolds for neural interfaces.

In addition, template‐assisted fabrication of PCL nanotube arrays has been used to build three‐dimensional (3D) scaffold components that mimic the nanoscale topography of bone tissue, improving mechanical strength and osteoblast adhesion (Abedalwafa et al. 2013). PLGA nanofibers can also be used as templates for the electrochemical deposition or coating of conductive polymers (Heo et al. 2025), such as poly(3,4‐ethylenedioxythiophene), which gives neural tissue engineering scaffolds with excellent electrical conductivity, biocompatibility, and ability to promote neural regeneration (Figure 7).

## 6 Function‐Oriented Design and Research‐Gap Map for Hybrid Nanotube Biomaterials

To further operationalize the “structure–property–bioresponse–function” framework proposed in this review, this section constructs a function‐oriented design and research‐gap map for future hybrid nanotube biomaterials. The preceding sections discussed representative nanotube systems in terms of biocompatibility, degradability, and biomedical applications. Future nanotube biomaterials with translational potential, however, are unlikely to rely indefinitely on single‐component systems and are more likely to employ hybrid or composite structures in which different basic material chemistries provide complementary functions. This section therefore first deconstructs representative nanotube systems into their basic material chemistries and summarizes the dominant functional contribution of each material class (Table 4). Pairwise functional intersections are then mapped to distinguish reported hybrid functional directions from potential gaps that still lack direct experimental validation and merit future investigation.

**TABLE 4:** Basic material chemistries and their dominant functional contributions.

Basic material chemistry	Representative nanotube systems	Dominant functional contribution
Carbon‐based materials	Carbon nanotube‐like systems	Electrical/optical signal transduction and mechanical reinforcement
Boron nitride materials	Boron nitride nanotube‐like systems	Piezoelectric energy conversion and boron‐related functionality
Titanium‐based materials	TiO_2_ nanotubular arrays	Implant‐interface modulation and local reservoir function
Aluminosilicate materials	Halloysite nanotube‐like systems	Lumen‐based loading and differential inner–outer surface functionalization
Alumina materials	Anodic alumina nanochannels	Ordered nanochannel templating and pore regulation
Lipid‐based materials	Lipid nanotube‐like systems	Biointerface compatibility and biodegradable delivery
Polymeric materials	Polymeric nanotube‐like systems	Structural programmability and degradation/flexibility modulation

Combinations 1–6 in Table 5 represent carbon‐centered hybrid nanotube systems. In these systems, carbon‐based tubular materials, most commonly represented by CNTs, provide electrical/optical signal transduction and mechanical reinforcement, while the second component contributes complementary functions such as stability, interface modulation, lumen‐based loading, pore regulation, biointerface compatibility, or structural programmability. The current evidence pattern indicates, however, that carbon‐centered hybrid nanotubes remain more developed in materials science than in biomedicine. Many reported combinations were originally designed for thermal management, photocatalysis, electrochemistry, adsorption, ceramic reinforcement, dispersion improvement, or composite strengthening rather than for regulating cell behavior, reducing inflammation, improving hemocompatibility, enabling safe degradation, or achieving predictable metabolic clearance. Their biological relevance therefore often remains indirect or insufficiently validated even when the combinations have been experimentally explored. This limitation is especially important for carbon‐centered systems because they remain strongly affected by CNT‐related translational barriers. CNTs, the most representative carbon‐based nanotube model, are not chemically or structurally uniform: synthesis can leave metal‐catalyst residues and carbonaceous impurities while generating broad variations in length, diameter, wall number, chirality, defect density, and aggregation state. These purity and scale‐heterogeneity issues make biological responses difficult to reproduce. In addition, the stable graphitic framework raises concerns regarding persistence, degradation, and clearance in vivo. Hybridization with boron nitride, TiO_2_, aluminosilicate, alumina, lipid, or polymeric components may improve selected physicochemical properties, but it does not automatically eliminate the purity, heterogeneity, persistence, and biosafety problems that constrain the translation of carbon‐based nanotube biomaterials.

**TABLE 5:** Functional‐combination evidence matrix for heterogeneous hybrid nanotube‐based biomaterials.

No.	Material combination	Dominant function from material A	Dominant function from material B
1	Carbon‐based materials + Boron nitride materials	Electrical/optical signal transduction and mechanical reinforcement (Jing, Samani, et al. 2017; Jing et al. 2016)	Piezoelectric energy conversion and boron‐related functionality (Choi et al. 2011)
2	Carbon‐based materials + Titanium‐based materials	Electrical/optical signal transduction and mechanical reinforcement (Vijayan et al. 2012; Cendrowski et al. 2014)	Implant‐interface modulation and local reservoir function (Hsieh et al. 2009)
3	Carbon‐based materials + Aluminosilicate materials	Electrical/optical signal transduction and mechanical reinforcement (MacKenzie and Bolton 2009)	Lumen‐based loading and differential inner–outer surface functionalization (Vanyorek et al. 2019)
4	Carbon‐based materials + Alumina materials	Electrical/optical signal transduction and mechanical reinforcement (Zhan et al. 2003)	Ordered nanochannel templating and pore regulation (Sankararamakrishnan et al. 2014)
5	Carbon‐based materials + Lipid‐based materials	Electrical/optical signal transduction and mechanical reinforcement (Gaunt et al. 2015)	Biointerface compatibility and biodegradable delivery (Gaunt et al. 2015; Zhao et al. 2021; Barbinta Patrascu et al. 2014)
6	Carbon‐based materials + Polymeric materials	Electrical/optical signal transduction and mechanical reinforcement (Ashrafi et al. 2017; Star et al. 2001)	Structural programmability and degradation/flexibility modulation (Zeng et al. 2010)
7	Boron nitride materials + Titanium‐based materials	Piezoelectric energy conversion and boron‐related functionality (Bustillos et al. 2020)	Implant‐interface modulation and local reservoir function
8	Boron nitride materials + Aluminosilicate materials	Piezoelectric energy conversion and boron‐related functionality (Kokulnathan et al. 2020)	Lumen‐based loading and differential inner–outer surface functionalization (Kokulnathan et al. 2020)
9	Boron nitride materials + Alumina materials	Piezoelectric energy conversion and boron‐related functionality (Wang et al. 2011)	Ordered nanochannel templating and pore regulation
10	Boron nitride materials + Lipid‐based materials	Piezoelectric energy conversion and boron‐related functionality	Biointerface compatibility and biodegradable delivery (Gupta et al. 2026)
11	Boron nitride materials + Polymeric materials	Piezoelectric energy conversion and boron‐related functionality (Ashrafi et al. 2017; Farshid et al. 2017)	Structural programmability and degradation/flexibility modulation (Farshid et al. 2017; Nithya and Pandurangan 2014; Emanet et al. 2016)
12	Titanium‐based materials + Aluminosilicate materials	Implant‐interface modulation and local reservoir function	Lumen‐based loading and differential inner–outer surface functionalization
13	Titanium‐based materials + Alumina materials	Implant‐interface modulation and local reservoir function (Lu et al. 2013)	Ordered nanochannel templating and pore regulation (Karp et al. 2007)
14	Titanium‐based materials + Lipid‐based materials	Implant‐interface modulation and local reservoir function	Biointerface compatibility and biodegradable delivery
15	Titanium‐based materials + Polymeric materials	Implant‐interface modulation and local reservoir function (Wang, Weng, et al. 2017)	Structural programmability and degradation/flexibility modulation (Wang, Weng, et al. 2017)
16	Aluminosilicate materials + Alumina materials	Lumen‐based loading and differential inner–outer surface functionalization	Ordered nanochannel templating and pore regulation
17	Aluminosilicate materials + Lipid‐based materials	Lumen‐based loading and differential inner–outer surface functionalization	Biointerface compatibility and biodegradable delivery
18	Aluminosilicate materials + Polymeric materials	Lumen‐based loading and differential inner–outer surface functionalization; related biomedical hybrid validation (Huang et al. 2019; Ou et al. 2020)	Structural programmability and degradation/flexibility modulation (Huang et al. 2019; Ou et al. 2020)
19	Alumina materials + Lipid‐based materials	Ordered nanochannel templating and pore regulation	Biointerface compatibility and biodegradable delivery
20	Alumina materials + Polymeric materials	Ordered nanochannel templating and pore regulation	Structural programmability and degradation/flexibility modulation
21	Lipid‐based materials + Polymeric materials	Biointerface compatibility and biodegradable delivery	Structural programmability and degradation/flexibility modulation

*Note:* Dark gray cells indicate directly validated hybrid biomaterial systems with experimental evidence supporting the defined functional combination. Light gray cells indicate related hybrid systems or non‐biological functional validation, for which the evidence supports material compatibility or partial functional coupling but not complete biomedical validation. Red cells indicate potential research gaps without direct experimental validation.

Combinations 7–11 correspond to boron‐nitride‐centered hybrid nanotube systems. Like carbon‐centered systems, boron‐nitride nanotubes face translational barriers related to purity, structural heterogeneity, dispersion, persistence, degradation, clearance, and long‐term biosafety; their functional logic nevertheless differs from that of carbon‐based systems. Carbon‐based nanotubes mainly serve as functional components for electrical/optical signal transduction, whereas boron‐nitride nanotubes are more likely to serve as structurally stable and electrically insulating tubular components. Their core value may therefore lie in structural–insulating integration: in highly miniaturized systems such as nanomachines or implantable micro/nanorobots, the nanotube wall may need to function simultaneously as a mechanical skeleton, protective shell, and insulating channel for internal conductive components. BN‐centered hybrid nanotubes may consequently be more suitable for scenarios requiring rigid, chemically stable, and electrically insulating tubular architectures than for conventional degradable delivery platforms.

Combinations 12–15 reflect the specific logic of titanium‐based hybrid nanotube systems. The key value of titanium‐based nanotubes does not lie in drug loading alone, because many nanotube platforms can provide lumen‐based storage; rather, their unique advantage derives from in situ integration with titanium or titanium‐alloy implants. Representative TiO_2_ nanotubular arrays are usually formed directly on titanium surfaces through controlled electrochemical oxidation–dissolution, creating substrate‐bound, vertically aligned, and ordered nanotubular interfaces. They are therefore better suited as local reservoirs and nanoscale tissue‐interfacing layers on titanium plates, screws, dental implants, or other orthopedic devices than as free‐standing nanotube building blocks for complex hybrid composites. The limited development of titanium‐based combinations with aluminosilicate, alumina, lipid, or polymeric materials may reflect a mismatch between the surface‐bound fabrication logic of titanium‐based nanotubes and the free‐standing or soft‐matter nature of many other nanotube systems. In this context, additional materials may be more useful as caps, coatings, or bioactive modifiers that regulate release and tissue responses than as equivalent structural components of free‐standing hybrid nanotube composites.

Combinations 16–18 reveal different degrees of evidence gaps among aluminosilicate‐centered hybrid nanotube systems. The representative aluminosilicate system, HNTs, already possesses a natural lumen and chemically distinct inner and outer surfaces and is intrinsically suitable for drug loading, surface modification, and controlled release. Combinations 16 and 17 still lack direct evidence demonstrating a clear additional biological value: aluminosilicate and alumina components both contribute mainly inorganic pore‐ or channel‐related functions and may therefore be functionally redundant, whereas lipid components are more commonly used as coatings, caps, or release‐regulating layers than as tubular bodies equivalent to HNTs. In contrast, Combination 18 has already undergone biomedical validation in polymeric hybrids such as HNT/GelMA systems. These studies show that the polymer matrix can provide a formable three‐dimensional network and cellular microenvironment, while HNTs enhance mechanical performance, provide nanotopography, and support osteogenesis, antibacterial activity, and immunomodulation (Huang et al. 2019; Ou et al. 2020). Existing work has not yet fully exploited the HNT lumen and the distinction between inner and outer surfaces for compartmentalized loading or multistage functional output. This combination is therefore better regarded as a direction with preliminary biomedical validation but incomplete functional coupling rather than as a complete research gap. Batch‐dependent variation in the purity, length, lumen size, surface charge, and mineral composition of naturally derived HNTs, together with uncertainties in degradation, clearance, and long‐term biosafety, remains a critical translational challenge.

Combinations 19–20 illustrate why alumina‐centered systems rarely develop into independent biomedical nanotube directions. Alumina nanochannels, particularly anodic‐alumina systems, are used primarily as ordered templates or nanochannel platforms rather than as intrinsically bioactive nanotube materials. Their principal strengths are highly ordered pore architecture, tunable pore size, structural stability, and templating capacity. When lipids or polymers are introduced, they are usually employed as coatings, sealing layers, pore modifiers, or release‐regulating components on alumina templates rather than as equivalent nanotube components that form a new hybrid nanotube system. Alumina/lipid and alumina/polymer combinations are therefore unlikely to emerge as independent biomedical nanotube directions. Their future value may depend on redefining alumina nanochannels not merely as passive templates but as ordered release pathways capable of guiding the spatial delivery and assembly of soft or bioactive materials.

Combination 21 represents the lipid/polymer soft‐material intersection. Its limited development as a hybrid nanotube direction may reflect the maturity of conventional lipid–polymer soft biomaterials. Lipid–polymer systems can already provide biocompatible interfaces, drug loading, controlled release, flexibility, and degradability in the form of nanoparticles, vesicles, micelles, coatings, membranes, or hydrogels; organizing both components into paired nanotube architectures therefore often lacks a clear necessity. Unless tubular geometry provides functions that conventional lipid–polymer formats cannot achieve, such as directional transport, spatially confined release, or tubular‐interface guidance, these systems are more likely to remain soft‐biomaterial platforms than to develop into independent hybrid nanotube biomaterials.

## 7 Clinically Oriented Assessment and Tiered Surveillance–Intervention Framework for Nanotube Applications

This framework evaluates candidate nanotube systems in their final application state, including modified, doped, composite, coated, and other engineered forms. Material‐related risk is not determined by material name alone, but should be assessed in relation to systemic exposure, biodistribution, clearance, long‐term persistence, and the tolerance of the patient and the specific application context (Bourquin et al. 2018; Yokel et al. 2012). The framework uses the Clinical Risk Score and Therapeutic Necessity Score as parallel inputs. A preliminary grade is generated through a risk–necessity matrix and then adjusted according to non‐compensable conditions, ultimately yielding Grade I–IV clinical surveillance and intervention strategies. The proposed scores, thresholds, and grade mappings are conceptual decision‐support settings and have not been clinically validated.

### 7.1 Step 1: Clinical Risk Score and Therapeutic Necessity Score

The four indicators respectively capture exposure extent, risk duration, patient tolerance, and clinical controllability after an abnormal event (Table 6). Systemic exposure is associated with whole‐body distribution and accumulation in the liver and spleen (Bourquin et al. 2018); residence time should be compatible with the therapeutic time window (Lucas et al. 2008; Zhang et al. 2014); baseline physiological reserve affects the patient's ability to tolerate material‐related inflammation, organ burden, and repeat intervention (Fried et al. 2001; Prigerson et al. 2015); and, for removable implants or device‐based systems, the feasibility of intervention or removal after an abnormal event directly affects risk control (Weise et al. 2005).

**TABLE 6:** Clinical risk score.

Code	Assessment item	0 points	1 point	2 points
R1	Systemic exposure	Predominantly confined to the local site	Limited systemic exposure	Definite entry into the systemic circulation or potential for widespread migration
R2	Compatibility between residence time and treatment timescale	Generally compatible	Partially incompatible	Clearly incompatible
R3	Baseline physiological reserve and treatment tolerance	Good baseline functional status, without major comorbidities or important organ dysfunction expected to impair tolerance	Some functional limitation, comorbidity, or reduced organ reserve that may increase material‐related risk	Markedly impaired physiological reserve, major organ dysfunction, or other conditions that substantially limit treatment tolerance
R4	Feasibility of intervention or removal after an abnormal event	Readily amenable to intervention or removal	Partially amenable to intervention or removal	Essentially not amenable to intervention or removal

*Note:* Total score and risk level: R1 + R2 + R3 + R4 = ____. A score of 0–2 indicates low clinical risk, 3–5 indicates moderate clinical risk, and 6–8 indicates high clinical risk.

The Therapeutic Necessity Score is not a material‐performance score. It integrates expected therapeutic benefit, the availability of acceptable alternatives, and dependence on nanotube‐related functions to determine whether the potential benefit is sufficient to justify material‐related risk and uncertainty (Table 7; Mühlbacher et al. 2016). When a mature, lower‐risk alternative with comparable efficacy is already available, the therapeutic necessity of the candidate nanotube system should be reduced accordingly (Suchard et al. 2019).

**TABLE 7:** Therapeutic necessity score.

Code	Assessment item	0 points	1 point	2 points
N1	Expected therapeutic benefit	Limited benefit	Clear benefit	Potential to substantially improve key therapeutic outcomes
N2	Availability of acceptable alternatives	A mature alternative with comparable effectiveness is available	Alternatives are available, but their effectiveness or applicability is limited	No effective or acceptable alternative is available
N3	Dependence on nanotube‐related functions	The target function does not require a nanotube system	The nanotube system can substantially enhance the target function but is not essential	The key therapeutic function is highly dependent on the nanotube structure or its engineered functions

*Note:* Total score and therapeutic necessity level: N1 + N2 + N3 = ____. A score of 0–2 indicates low therapeutic necessity, 3–4 indicates moderate therapeutic necessity, and 5–6 indicates high therapeutic necessity.

### 7.2 Step 2: Integrated Decision

The clinical risk level and therapeutic necessity level obtained from Tables 6 and 7 are entered into the risk–necessity matrix to determine a preliminary grade (Table 8).

**TABLE 8:** Risk–necessity matrix.

Clinical risk level	Low therapeutic necessity	Moderate therapeutic necessity	High therapeutic necessity
Low clinical risk	Grade I	Grade I	Grade I
Moderate clinical risk	Grade IV	Grade II	Grade II
High clinical risk	Grade IV	Grade IV	Grade III

*Note:* 1. This matrix is a conceptual decision‐support tool rather than a validated clinical prediction model. 2. The matrix outputs a clinical management grade, not a recommendation to use the candidate system. Under low clinical risk, the therapeutic necessity level does not alter the intensity of basic surveillance. Grade I indicates only that, if the system is used, it may be incorporated into basic clinical management; whether a sufficient clinical indication exists must still be determined independently. Adjustment note: Table 8 provides only a preliminary grade. The system should be assigned to Grade IV regardless of the preliminary result if either of the following non‐compensable conditions is present: clinically unacceptable long‐term persistence without a clearly defined clearance pathway; or a highly persistent candidate system that lacks both reliable material traceability and a clinically feasible intervention pathway (Bourquin et al. 2018; Yokel et al. 2012). Grade III additionally requires reliable material traceability and a predefined, clinically feasible intervention pathway; otherwise, it should be adjusted to Grade IV.

### 7.3 Step 3: Tiered Clinical Surveillance and Intervention Strategies

Grades I–IV represent different levels of clinical management rather than fixed treatment protocols (Table 9). Surveillance design should follow the principles of risk‐adapted follow‐up, sequential comparison, and threshold‐triggered intervention (Brady et al. 2004; Schoots and Padhani 2020; Imhoff and Kuhls 2006; van Rossum et al. 2022).

**TABLE 9:** Tiered clinical surveillance and intervention strategies.

Grade	Clinical management position	Surveillance strategy	Intervention strategy
Grade I	Basic clinical observation	Select basic assessment items according to the expected in vivo fate, application site, and major risks of the candidate nanotube system, and incorporate them into the existing treatment follow‐up pathway.	For mild, transient, or nonspecific abnormalities, prioritize low‐burden, non‐invasive measures; adjust the treatment plan or follow‐up schedule when necessary.
Grade II	Enhanced screening and abnormality detection	Integrate material‐related screening into routine examinations, scheduled reviews, or disease follow‐up. Use low‐burden, repeatable assessments to identify persistent changes or abnormal signals; abnormal signals trigger further diagnostic evaluation.	Provide targeted treatment according to the confirmed type, severity, and progression of the abnormality, and adjust subsequent surveillance intensity accordingly.
Grade III	Traceable surveillance and threshold‐triggered intervention	Before application, establish a reliable material‐tracking plan and risk‐related baseline, including inflammatory status, material location and distribution, relevant functional status, and monitorable structural or performance characteristics. After application, conduct sequential comparisons and monitor predefined abnormality thresholds.	Predefine the correspondence between abnormal events and management measures. Once a specified threshold is reached, initiate the corresponding pharmacological treatment, interventional procedure, repeat surgery, removal, replacement, or other predefined intervention pathway.
Grade IV	Defer or do not recommend application	—	Defer, discontinue, or do not recommend clinical application. Prefer more mature, lower‐risk, or more controllable alternatives, and reassess only after substantial improvement in key risk conditions, traceability, or intervention feasibility.

*Note:* Grades I–III follow a cumulative escalation principle, meaning that each higher grade includes the basic surveillance and intervention measures of the preceding grade. Grade IV represents a decision to defer or not recommend application rather than a further increase in surveillance intensity. Specific items should be selected and combined according to the actual in vivo fate, application site, anticipated risk pattern, and individual patient characteristics of the candidate nanotube system.

Overall, this framework integrates material persistence, exposure extent, patient tolerance, therapeutic necessity, and post‐abnormality intervention feasibility within a single decision pathway. It is intended to provide a conceptual reference for subsequent preclinical validation, risk communication, and tiered management, rather than to replace indication‐specific judgment or validated clinical decision tools.

## 8 Conclusion

This review has provided a comprehensive overview of biocompatibility, degradability, and biomedical applications of various nanotube systems with great potential in medicine.

The biocompatibility of nanotubes is determined by their surface chemistry, morphology, and purity, which collectively determine their interactions with biological systems. Surface functionalization strategies, such as the introduction of reactive groups or polymer coatings, can enhance both biocompatibility and, in some cases, biodegradability, establishing the first link between structural design and biological performance. The degradability and metabolic fate of nanotubes depend mainly on the bond type and chemical reactivity, which relate their intrinsic physicochemical stability to their biological behavior.

As drug carriers, nanotubes achieve high loading capacity, targeted delivery, and controlled release, improving therapeutic efficacy while minimizing systemic toxicity. As imaging and diagnostic platforms, their optical, electrical, magnetic, and structural features enable enhanced sensitivity and multimodal contrast. As tissue‐engineering scaffolds, their exceptional mechanical strength and tuneable surface characteristics provide favorable microenvironments for cell adhesion, proliferation, and differentiation, thereby facilitating tissue repair and regeneration.

Beyond these shared principles, different nanotube systems occupy distinct positions within the “structure–property–bioresponse–function” framework: CNTs, with their outstanding electrical and thermal conductivity, are well suited to bioimaging and sensing; BNNTs combine chemical stability and piezoelectric responsiveness and show potential in bone regeneration and neutron‐capture therapy; TiNTs exhibit excellent osseointegration and antibacterial activity and are suitable for implant‐surface modification; and HNTs, LNTs, and PNTs offer favorable biocompatibility and degradability as carriers for sustained drug release.

Taken together, these findings highlight that nanotubes should not be considered isolated materials, but rather a platform of tuneable materials whose medical performance derives from the interplay between structure, physicochemical properties, and biological interactions. Understanding and controlling this interaction will be essential to bridge the gap between material design and clinical application, paving the way for multifunctional nanotube‐based systems that integrate diagnosis, therapy, and regeneration in next‐generation biomedicine.

## Author Contributions

**Yue Zhong:** conceptualization, visualization, writing – original draft, writing – review and editing. **José A. Lebrón:** visualization, supervision, writing – original draft, writing – review and editing. **Manuel López‐López:** visualization, writing – original draft, resources, conceptualization, supervision, writing – review and editing, project administration. **Pilar López‐Cornejo:** visualization, supervision, writing – original draft, writing – review and editing, resources, project administration. **María L. Moyá:** visualization, writing – original draft, resources. **Francisco J. Ostos:** conceptualization, visualization, supervision, writing – original draft, writing – review and editing, resources, project administration.

## Funding

This work was financed by the Consejería de Universidad, Industria, Energía e Innovación de la Junta de Andalucía (FQM‐206, FQM‐274); the Consejería de Salud y Consumo de la Junta de Andalucía (PI‐0170‐2024); the Ministerio de Ciencia, Innovación y Universidades (PID2023‐151642OB‐I00); the European Union (FEDER funds); the Cátedra Fertinagro Biotech de la Universidad de Huelva (NANOCARB and NAPABA); and by the University of Seville (Ayudas Servicios Generales de la Universidad, CITIUS, 2025/00000399, 2025/00000418). The authors acknowledge the contract of Y. Zhong supported by Guizhou Junzang Testing Service Co. Ltd. (2024, Enterprise‐Sponsored Project).

## Conflicts of Interest

The authors declare no conflicts of interest.

## Data Availability

Data sharing is not applicable to this article as no new data were created or analyzed in this study.
